# Quantum Broadcast Channel Simulation via Multipartite Convex Splitting

**DOI:** 10.1007/s00220-024-05191-4

**Published:** 2025-01-16

**Authors:** Mario Berta, Hao-Chung Cheng, Li Gao

**Affiliations:** 1 Institute for Quantum Information, RWTH Aachen University, Aachen, Germany; 2 Department of Electrical Engineering and Graduate Institute of Communication Engineering, National Taiwan University, Taipei 106319, Taiwan; 3 Department of Mathematics, National Taiwan University, Taipei, 106319 Taiwan; 4 Center for Quantum Science and Engineering, National Taiwan University, Taipei, 106319 Taiwan; 5 Physics Division, National Center for Theoretical Sciences, Taipei, 106319 Taiwan; 6 Hon Hai (Foxconn) Quantum Computing Center, New Taipei City, 236, Taiwan; 7 School of Mathematics and Statistics, Wuhan University, Wuhan, 430072 China

## Abstract

We show that the communication cost of quantum broadcast channel simulation under free entanglement assistance between the sender and the receivers is asymptotically characterized by an efficiently computable single-letter formula in terms of the channel’s multipartite mutual information. Our core contribution is a new one-shot achievability result for multipartite quantum state splitting via multipartite convex splitting. As part of this, we face a general instance of the quantum joint typicality problem with arbitrarily overlapping marginals. The crucial technical ingredient to sidestep this difficulty is a conceptually novel multipartite mean-zero decomposition lemma, together with employing recently introduced complex interpolation techniques for sandwiched Rényi divergences. Moreover, we establish an exponential convergence of the simulation error when the communication costs are within the interior of the capacity region. As the costs approach the boundary of the capacity region moderately quickly, we show that the error still vanishes asymptotically.

## Introduction

*Quantum channel simulation* endeavors to simulate a noisy quantum channel via a limited resource of noiseless channels and potentially other assistance such as, e.g., free entanglement. For simulating a point-to-point channel ${\mathscr {N}}_{A\rightarrow B}$ in the independent and identically distributed (i.i.d.) setting, Bennett et al. showed that under free entanglement-assistance the amount of asymptotic classical communication rate needed is equal to the quantum mutual information of channel [3, 3, 3]

1.1 $$\begin{aligned} I({\mathscr {N}}_{A\rightarrow B}) \,{:=}\, \sup _{\rho } I(B:R)_{({\mathscr {N}}\otimes \textrm{id})\left( \psi ^\rho \right) }, \end{aligned}$$

where the maximum ranges over all input states $\rho _{A}$, $\psi _{AR}^\rho$ is a purification of $\rho _A$ by a reference system *R*, and *I* denotes the quantum mutual information. Notably, the aforementioned classical communication cost coincides with the entanglement-assisted classical channel capacity [5, 5, 5]. Hence, from both operational and information-theoretic perspectives, point-to-point quantum channel simulation may be viewed as a *reverse* task of the quantum version of Shannon’s celebrated noisy coding theorem for simulating a noiseless channel via a noisy channel [6]. Accordingly, such a result is also termed the *Quantum Reverse Shannon Theorem*.

At first glance, one may wonder why to lavish noiseless channels on simulating the noisy one. To answer this question, firstly, the noisy coding theorem in conjunction with the Quantum Reverse Shannon Theorem lead us to characterizing *resource inter-conversion* [7, 8], e.g. finding the capacity of simulating a channel ${\mathscr {N}}$ from an arbitrary channel ${\mathscr {M}}$ in the presence of pre-shared entanglement in terms of a single quantity. Secondly, for the classical and classical-quantum special case, the channel simulation task may date back to the earlier studies on correlation generations [8–14], and channel resolvability [13, 15–23], which deliver applications to wiretap channel coding [24–28], communication complexity of correlation [29], and measurement compression [30, 31, 33, 33]. Further information-theoretic applications include strong converse theorems [30, 30, 30], lossy data compression [30, 34–36], local purity distillation [39, 39, 39, 40], and entanglement distillation [41]. We refer the readers to [3, 14, 14, 14, 14] for more comprehensive reviews on this topic. It is worth mentioning that many of the above-mentioned works presume the input source to the channel to be fixed. Nonetheless, we will consider channel simulation under arbitrary sources; that is, we aim for a channel simulation result that works for the worst-case input scenario.

Notwithstanding the recent advances on point-to-point quantum channel simulation [3, 43–45], the asymptotic capacity region of general *quantum broadcast channel* simulation was left unknown prior to the present work. Broadcast channels [46–51] are arguably one of the most simplest and practical network models in multi-user coding settings. This setting comprises a base station that wants to transmit down-link information to numerous and separate user equipments that only have their own local decodability.^1^ However, despite considerable efforts, the capacity region for a two-receiver broadcast channel coding is still unknown.^2^ On the reverse side of channel simulation, somewhat surprisingly, the capacity region for *classical* broadcast channel simulation under common randomness-assistance has recently been characterized in [61]. This naturally leads to a question: “Can one obtain the capacity region for *quantum* broadcast channel simulation under free entanglement-assistance?”

Unfortunately, multi-user tasks are challenging in the fully quantum setting, since they are closely related to a serious technical barrier, the so-called *quantum joint typicality* [61] or *simultaneous smoothing conjecture* [62], and more generally the *quantum marginal problem* [63]. Consequently, previously only specialized scenarios of broadcast channel simulation could be handled. This includes the fixed i.i.d. input case via time sharing methods [64, 65], isometric broadcast channels [66] (see also [65]), and the recent result [67] on classical broadcast channels.

**Fig. 1:**
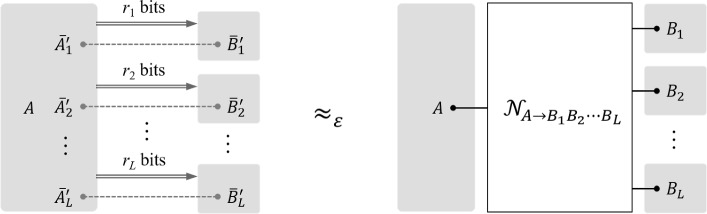
Depiction of an *L*-receiver quantum broadcast channel simulation. The Sender holds system *A* and share free entanglement with *L* receivers. By sending $r_1$, $r_2$, $\ldots$, $r_L$ bits of classical information to each receiver and local quantum operation at each receiver, the resulting transformation is close to an *L*-receiver quantum broadcast channel ${\mathscr {N}}_{A\rightarrow B_1B_2\ldots B_L}$ (in diamond norm). Note that each gray-shaded region is only allowed to perform local quantum operation

In this paper, we establish the capacity region of general quantum broadcast channel simulation under free entanglement-assistance by circumventing the aforementioned obstacles around the quantum joint typicality conjecture. Taking the two-receiver broadcast channel ${\mathscr {N}}_{A\rightarrow B_1 B_2}$ as an example, we show that the channel simulation is asymptotically achievable if and only if the classical communication costs $r_1$ and $r_2$ from the sender to each of two receivers (see Fig. 1) satisfy

1.2 $$\begin{aligned} \left\{ (r_1, r_2) \in \mathbb {R}^2: r_1 + r_2 \ge I({\mathscr {N}}_{A\rightarrow B_1 B_2} ), \, r_1 \ge I( {\mathscr {N}}_{A\rightarrow B_1}), \, r_2 \ge I( {\mathscr {N}}_{A\rightarrow B_2} ) \right\} , \end{aligned}$$

where the sum-rate is constrained by the *bipartite mutual information* of channel ${\mathscr {N}}_{A\rightarrow B_1 B_2}$ [67, 68] [see the precise definition in Eq. (2.13)]. Notably, we do not rely on the time-sharing technique and the capacity region for arbitrary *L* receivers can be obtained as well (Theorem 8).^3^ Our proof techniques build on a conceptually new version of the *multipartite convex-split lemma*, a corresponding *multipartite Quantum State Splitting* protocol, and the (by now standard) *Post-Selection Technique* [69]. As our approach gets around a generic instance of the quantum joint typicality conjecture with arbitrarily overlapping marginals, we believe that it may serve as a general recipe for fully quantum network information-theoretic tasks.

**Fig. 2:**
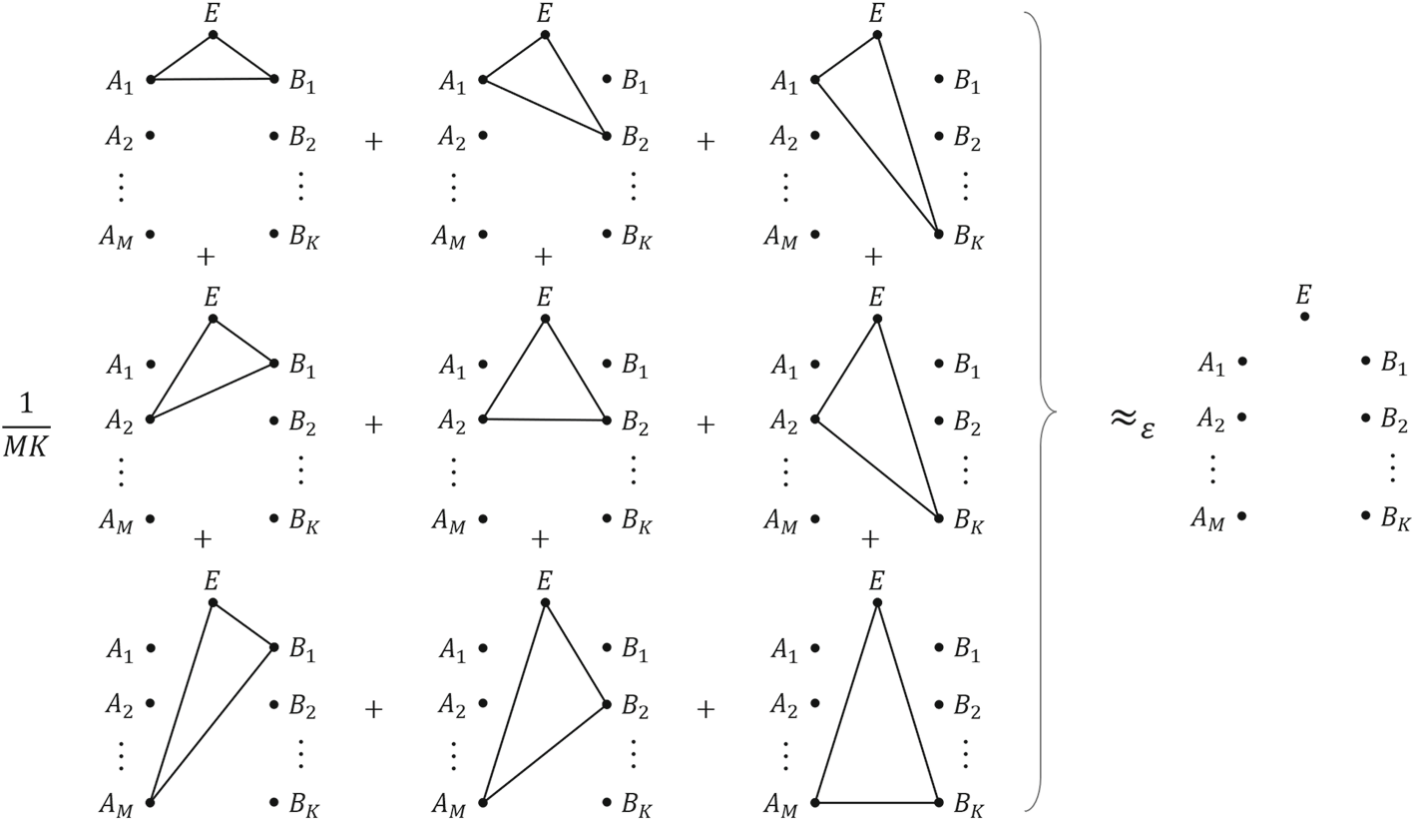
Depiction of a bipartite convex splitting. On the right part, each isolated dot represents an independent quantum system, and hence the overall state is product. On the left part, solid lines connected each system is a tripartite (correlated) state, while the other systems are left isolated. When *M* and *K* are sufficiently large (see Theorem 1), then the statistical mixture of the left part is close to the right part of product state (in trace norm)

*Convex splitting* was introduced by Anshu et al. in [70] (see also [71, 72]), which originates from the idea of *rejection sampling* in statistics [73–75], and it has an ample of applications in quantum information theory. In this paper, we generalize it to a conceptually new multipartite version and establish an one-shot error exponent bound.^4^ Taking the bipartite version as an illustration, assume that there are *M* independent and identical copies of registers *A*’s, *K* copies of registers *B*’s, and a single register *E*. Now the overall system is prepared in a way that register *E* is correlated to the *m*-th register *A* and the *k*-th register *K* uniformly at random (see Fig. 2). We then prove a tight one-shot upper bound on the trace distance between such a joint state and the all-tensor-product state (i.e. all of the systems *A*’s, *B*’s, and *E* are decoupled) in terms of a generalized quantum Rényi information [76–78] (Theorem 1). The additivity of the Rényi information then immediately gives us a rate region for *M* and *K*, for which the trace distance decreases exponentially in the i.i.d. asymptotic limit. This thus can be viewed as a bipartite generalization of the unipartite convex splitting by part of the authors [72]. To establish the result and avoid the need of the simultaneous smoothing, we introduce a key ingredient of a decomposition map, the *multipartite mean-zero decomposition lemma* (Lemmas 3 & 5).^5^ We remark that a similar idea is independently proposed by Colomer Saus and Winter for deriving multipartite quantum decoupling theorems, termed the *telescoping trick* in their work [80].

An immediate application of the multipartite convex-split lemma is the multipartite version of *Quantum State Splitting* [64, 65] also termed *mother protocol* in its original (non-multipartite) form [81]. The goal is to transfer the systems $A_1'$, $A_2'$, $\ldots$, $A_L'$ initially with Alice, to Bob, while the entanglement between all of Alice’s original systems *A* and an inaccessible reference system *R* is preserved. Given any classical communication cost consumed in the protocol, we obtain an one-shot error exponent bound on how well the protocol is performed.

Armed with multipartite Quantum State Splitting, we demonstrate how to combine the Post-Selection Technique [69] (as used in point-to-point quantum channel simulation [3]) and the quantum sandwiched information (Lemmas 1 and 2), to establish a one-shot error exponent bound for multipartite quantum broadcast channel simulation (Theorem 7) with diamond norm [82, 83] as an error criterion. We note that such a one-shot bound not only leads to the optimal achievable rate region in the i.i.d. setting; it also guarantees that the error of channel simulation decreases exponentially fast in the number of blocklength *n* whenever the rate vector of communication costs is in the interior of the capacity region. Furthermore, we show the achievability part of a *moderate deviation* result [84, 85]. Namely, the error of simulation will vanish asymptotically, even though the rate vector converges to the boundary of the capacity region at a moderate speed (Proposition 4).

Last but not least, let us point out some distinctive features of our results. The established multipartite convex splitting (Theorem 2) and multipartite Quantum State Splitting (Theorem 4) are *one shot*, in the sense that no mathematical constraint such as the i.i.d. assumption is needed. As for quantum broadcast channel simulation, we require the i.i.d. structure for the underlying channel to simulate. Our result (Theorem 7) is therefore *non-asymptotic* and for *any* finite blocklength, i.e. the assumption of infinitely large blocklength is not required. The reason behind our results is that we do not employ *time-sharing techniques* [47, Proposition 4.1], as this is not possible for the one-shot or finite blocklength setting (as pointed out even in classical network information theory [86, Remark 3]; see also [87]). By its nature, we believe that the proposed one-shot analysis, and in particular the multipartite *mean-zero decomposition lemma* (Lemma 5), could be a generic solution to problems in quantum network information theory.

The paper is structured in the following. We present an overview of our technical results in Sects. 1.1, and 1.2 contains discussions of related work. Notation and definitions for information measures are introduced in Sect. 2. Section 3 is devoted to establishing the multipartite convex splitting. The multipartite Quantum State Splitting achievability result is derived in Sect. 4. We give the proof of quantum broadcast channel simulation in Sect. 5. Some technical lemmas are left to the Appendices A and B.

### Overview of results

Our results are summarized below.

1. *Multipartite convex splitting*: For readability, we first present the result of the bipartite convex splitting. Let $\rho _{ABE}$, $\tau _A$, $\tau _B$ be states and let *M* and *K* be integers. The trace distance (i.e. trace norm divided by two) between the random mixture
  1.3 $$\begin{aligned} \frac{1}{MK}\sum _{m,k}\rho _{A_{m} B_{k}E} \bigotimes _{ \bar{m} \ne m, \, \bar{k} \ne k } \tau _{A_{\bar{m}}} \otimes \tau _{B_{\bar{k}}} \end{aligned}$$
  and the product state $\tau _{A}^{\otimes M} \otimes \tau _{B}^{\otimes K} \otimes \rho _E$ is upper bounded by (Theorem 1)
  1.4 $$\begin{aligned} 2\cdot \,2^{ - E_{\log MK}(\rho _{ABE} \,\Vert \, \tau _A \otimes \tau _B ) } + 2^{- E_{\log M}(\rho _{AE} \,\Vert \, \tau _A )} + 2^{- E_{\log K}(\rho _{BE} \,\Vert \, \tau _B )}, \end{aligned}$$
  where the error-exponent function
  1.5 $$\begin{aligned} E_r(\rho _{AE}\,\Vert \, \tau _A) \,{:=}\, \sup _{\alpha \in [1,2]} \frac{\alpha -1}{\alpha }\left( r - I_\alpha (\rho _{AE}\,\Vert \, \tau _A)\right) \end{aligned}$$
  is the Fenchel–Legendre transform of the Rényi information [78]
  1.6 $$\begin{aligned} I_\alpha (\rho _{AE}\,\Vert \, \tau _A)\,{:=}\, \inf _{\sigma _E} D_\alpha (\rho _{AE}\,\Vert \, { \tau _A } \otimes \sigma _E) \end{aligned}$$
  and $D_\alpha (\rho \,\Vert \,\sigma ) \,{:=}\, \frac{1}{\alpha -1} \log {{\,\textrm{Tr}\,}}\left[ (\sigma ^{\frac{1-\alpha }{2\alpha }} \rho \sigma ^{\frac{1-\alpha }{2\alpha }} )^\alpha \right]$ is the quantum sandwiched Rényi divergence [76, 77]. Moreover, the three error exponents in Eq. (1.4) are all positive if and only if
  1.7 $$\begin{aligned} {\left\{ \begin{array}{ll} & \log MK> I_1(\rho _{ABE} \,\Vert \, \tau _A \otimes \tau _B ) \\ & \log M> I_1(\rho _{AE} \,\Vert \, \tau _A )\\ & \log K > I_1(\rho _{BE} \,\Vert \, \tau _B )\\ \end{array}\right. }. \end{aligned}$$
  This then gives us an achievable rate region for a bipartite convex splitting. The result generalizes to arbitrary *L*-party case. Let $\rho _{A_1 A_2\ldots A_L E}$ and $\tau _{A_{\ell }}$ be states and $M_\ell$ be an integer for each $\ell \in \{1,2,\ldots , L\} =: [L]$. The trace distance between the random mixture
  1.8 $$\begin{aligned} \frac{1}{M_1 \cdots M_L}\sum _{m_\ell \in [M_\ell ], \, \ell \in [L]}\rho _{A_{ 1, m_1} \cdots A_{ L, m_L} E}\bigotimes _{ \bar{m}_{\ell } \in [M_{\ell }]/\{m_\ell \}, \, \ell \in [L] } \tau _{ A_{\ell , \bar{m}_{\ell } } } \end{aligned}$$
  and the product state $\bigotimes _{\ell \in [L]} \tau _{ { A_{\ell } } }^{\otimes M_\ell } \otimes \rho _E$ is upper bounded by (Theorem 2)
  1.9 $$\begin{aligned} \sum _{\varnothing \ne S\subseteq [L] } 2^{|S|} \cdot 2^{ - E_{\log M_S}\left( \rho _{A_S E} \,\Vert \, \tau _{A_S}\right) }, \end{aligned}$$
  where $A_S$ denotes systems $A_\ell$ for all $\ell \in S$, $\tau _{A_S }\,{:=}\, \bigotimes _{\ell \in S } \tau _{A_\ell }$, and $M_S \,{:=}\, \prod _{\ell \in S} M_\ell$. Again, the achievable rate region is given by
  1.10 $$\begin{aligned} \left\{ (M_1,M_2, \ldots , M_L) : \log M_S > I_1( { \rho _{A_S E} } \,\Vert \, \tau _{A_S} ), \, \forall S\subseteq [L] \right\} . \end{aligned}$$
2. *Multipartite Quantum State Splitting*: Let $\rho _{AA_1'A_2'\ldots A_L' R}$ be a pure state holding by Alice and an inaccessible reference system *R*. Suppose that Alice and the $\ell$-th Receiver share many-copies of entangled state $|\tau \rangle _{\bar{A}_\ell ' \bar{B}_\ell }$, where $\bar{A}_\ell ' \cong \bar{B}_\ell \cong A_\ell '$. The goal of an *L*-party Quantum State Splitting is to transfer each system $A_\ell '$ to the $\ell$-th Receiver via $r_\ell$ bits of classical communication. Then, the error in terms of trace distance is upper bounded by (Theorem 4)
  1.11 $$\begin{aligned} \sqrt{ \sum \nolimits _{\varnothing \ne S\subseteq [L] } 2^{|S|} \cdot 2^{ - E_{r_S}\left( \rho _{A_S' R} \,\Vert \, \tau _{A_S'}\right) }}, \end{aligned}$$
  where $r_S\,{:=}\, \sum _{ \ell \in S } r_\ell$. Moreover, the error exponents are all positive if and only if for all $S \subseteq [L]$,
  1.12 $$\begin{aligned} r_S > I_1\left( \rho _{A_S' R} \,\Vert \, \tau _{A_S'}\right) . \end{aligned}$$
3. *Quantum Broadcast Channel Simulation*: Consider a general *L*-receiver quantum broadcast channel ${\mathscr {N}}_{A \rightarrow B_1B_2\ldots B_L}$, and free entanglement is present between Sender and each of the *L* Receivers. By sending $n r_\ell$ bits of classical information to the $\ell$-th Receiver, respectively, the simulated channel is close to ${\mathscr {N}}_{A \rightarrow B_1B_2\ldots B_L}^{\otimes n}$ in diamond norm [82, 83] with error at most (Theorem 7)
  1.13 $$\begin{aligned} \varepsilon _n \le k_n \cdot \sqrt{ \sum \nolimits _{\varnothing \ne S \subseteq [L]} 2^{|S|} \cdot 2^{- n E_{r_S}({\mathscr {N}}_{A\rightarrow B_S}) }}, \end{aligned}$$
  where $B_S$ denotes the systems $B_\ell$ for all $\ell \in S$, and $k_n$ is a polynomial pre-factor depending on *L* and the dimension of the input space. The function $E_{r_S}({\mathscr {N}}_{A\rightarrow B_S})$ is the Fenchel–Legendre transform of $\sup _{\psi _{AR}} I_\alpha ({\mathscr {N}}_{A\rightarrow B_S}(\psi _{AR})\,\Vert \, \otimes _{\ell \in S} {\mathscr {N}}_{A\rightarrow B_{\ell }}(\psi _A))$ . Via a minimax identity (Proposition 2), it is equal to the error exponent corresponding to the worst-case purification $\psi _{AR}$. We remark that this result holds for *any* finite-blocklength *n*. The achievability result given in Eq. (1.13) leads us a lower bound on the overall error exponent (Proposition 3)
  1.14 $$\begin{aligned} \liminf _{n\rightarrow \infty } -\frac{1}{n} \log \varepsilon _n \ge \frac{1}{2}\min _{\varnothing \ne S\subseteq [L]} E_{r_S}\left( {\mathscr {N}}_{A\rightarrow B_S}\right) . \end{aligned}$$
  Then, we show that the capacity region of the broadcast channel simulation is given by (Theorem 8)
  1.15 $$\begin{aligned} \left\{ (r_1 , r_2, \ldots , r_L):\sum \nolimits _{ \ell \in S } r_\ell \ge I({\mathscr {N}}_{A\rightarrow B_S}), \forall S \subseteq [L]\right\} , \end{aligned}$$
  where the *multipartite quantum mutual information of channel* ${\mathscr {N}}_{A\rightarrow B_S}$ for a non-empty subset $S \subseteq [L]$ is defined as $I({\mathscr {N}}_{A\rightarrow B_S}) \,{:=}\, \sup _{\psi _{AR}} D\big ({\mathscr {N}}_{A\rightarrow B_S}( \psi _{AR} ) \,\Vert \otimes _{\ell \in S} {\mathscr {N}}_{A\rightarrow B_{\ell } }( \psi _{A} ) \otimes \psi _R \big )$, where the supremum is taking over all pure states $\psi _{AR}$ on system *AR*. The achievability in Eq. (1.13) also implies an achievability of the *moderate deviation* result [84, 85] as follows. For any non-empty subsets $S \subseteq [L]$, assume that the rates satisfy $\sum _{\ell \in S} r_{\ell ,n} - I({\mathscr {N}}_{A\rightarrow B_S}) = \Theta (n^{-t})$ for some $t\in (0, 1/2)$. That is, the *L*-tupe rate asymptotically converges to the boundary of the capacity region at certain speed. Then, we show that the error $\varepsilon _n$ of simulation still vanishes asymptotically (Proposition 4)
  1.16 $$\begin{aligned} \varepsilon _n \le O\left( 2^{- n^{1-2t} } \right) \rightarrow 0. \end{aligned}$$

### Related works

The time reversal of Quantum State Splitting corresponds to coherent *Quantum State Merging* [81]. Further, a variant thereof, termed *non-coherent* Quantum State Merging was proposed by Horodecki et al. in [88, 89]. For the latter, one gives free LOCC and quantifies the entanglement needed to achieve the task of Quantum State Merging, whereas for the former one gives free entanglement and quantifies the communication requirements. The multipartite version of non-coherent Quantum State Merging was already studied in [89] as well, which then corresponds to the task of fully quantum *distributed compression*. We note that this multipartite Quantum State Merging result relies on time-sharing techniques [47, Proposition 4.1], meaning that once the (simple) corner points of the capacity region are achieved, time sharing leads to the convex hull of them.^6^ Subsequently, the need of time sharing was removed for the 2-party setting [90, 61, §3.2.4]. However, it is unclear if their technique applies to more general multi-party setting and hence the quantum joint typicality conjecture is needed [61, Conjecture 3.2.7].^7^ A possible way to tackle the problem could be using the techniques from [62] and the minimax smoothing from [91] even to tight cost functions in terms of smooth conditional min-entropy. However, working instead with the coherent Quantum State Merging task, it is unclear to us how to transform these entanglement cost functions to communication cost functions, in a way that retains joint smoothing and allows to later apply the Post-Selection Technique for quantum broadcast channel simulation. In short, for multipartite coherent *Quantum State Merging*, one way is to solve an instance of the joint smoothing problem with overlapping marginals (c.f. [61, Conjecture 4.1.3]). On the other hand, Colomer Saus and Winter’s concurrent work to the present paper [80] is able to solve this problem by employing a technique with the same spirit to our *mean-zero decomposition lemma*, that we will introduce later in Lemmas 3 and 5.

The technique of (unipartite) *convex splitting* was introduced by Anshu et al. in [70, 71]. The idea originated from *rejection sampling* in statistics [73, 74, Chapter 2.3], [75], and one of its specialized cases dates back to the classical *soft covering* by Wyner et al. [11, 15, 16, 19, 23, 26, 27, 92–97]. It was recently generalized to an one-shot error-exponent result by parts of the authors [72]. The convex splitting under the measure of quantum relative entropy is also studied in Ref. [98]. The bipartite classical convex-split lemma - for which all the density operators share the same eigenbasis - was shown in [71, Fact 7]. Its straightforward generalization to the multipartite version was shown in [66, Lemma 39], which is the key lemma for showing classical broadcast channel simulation.

A bipartite *quantum* convex-split lemma was shown in [66, Lemma 2].^8^ However, it is not clear to us whether this would lead to the optimal achievable rate region in the i.i.d. setting. In fact, [66, Theorem 4] provides an asymptotic i.i.d. analysis with an assumption that the whole joint state, i.e. $\rho _{ABE}$ in Eq. (1.4), is pure. To the best of our knowledge, this will only give an *isometric broadcast channel simulation* (see also [65]), rendering the simulation of *general* quantum broadcast channels previously open. On the other hand, a “bipartite” convex-split lemma was also shown in [99], wherein the system *E* is absent. We remark that a multipartite convex-split lemma with the presence of system *E* is crucial for the multipartite Quantum State Splitting and quantum broadcast channel simulation since the system *E* plays the role of the reference system *R* with which we want to protect the entanglement.

Indeed, establishing a one-shot achievability lemma for bipartite or multipartite settings that will yield the right achievable rate region is the central problem in quantum network information theory [61–63, 89, 90, 101, 101]. Our breakthrough here is to introduce a mean-zero decomposition lemma (Lemmas 3 and 5) such that we can generally bypass the *simultaneous smoothing/interpolation* obstacles in the fully quantum setting. Taking the bipartite convex splitting as an example. The established one-shot bound in Eq. (1.4) immediately implies the achievable region given in Eq. (1.7). Hence, no sophisticated tools in asymptotic equipartition property such as typical projection onto subsystems, gentle measurement lemma [102], and the second-order asymptotics are needed. On the contrary, only the additivity of Rényi information is needed.

## Notation and Information Quantities

Throughout this paper, the underlying Hilbert spaces associated to quantum registers/systems *A*, *B*, *C*, $\ldots$, are denoted by sans-serif fonts ${\textsf{A}}$, ${\textsf{B}}$, ${\textsf{C}}$, $\ldots$, et cetera. The set of density operators (i.e. positive semi-definite operators with unit trace) and bounded operators on ${\textsf{A}}$ are denoted by ${\mathcal {S}}({\textsf{A}})$ and ${\mathcal {B}}({\textsf{A}})$, respectively. The notation $|{\textsf{A}}|$ stands for the dimension of Hilbert space ${\textsf{A}}$. For a density operator $\rho _A$ or a bounded operator $X_A$ with subscript *A*, we mean the operator is on the Hilbert space ${\textsf{A}}$ and often as the corresponding marginal of a multippartite operator *X*. We will use the term density operator and quantum state interchangeably in this paper. The logarithm is with base 2 throughout this paper.

For any $X \in {\mathcal {B}}({\textsf{A}})$, we define $\Vert X \Vert _p \,{:=}\, \left( {{\,\textrm{Tr}\,}}[|X|^p] \right) ^{1/p}$ for $p>0$. We use $\mathbb {N}$ and $\mathbb {R}$ to denote positive integers and real numbers. For any positive integer $M\in \mathbb {N}$, we shorthand the set $[M]\,{:=}\, \{1,2,\ldots , M\}$. We denote $\textrm{id}_R: {\mathcal {B}}({\textsf{R}}) \rightarrow {\mathcal {B}}({\textsf{R}})$ as the canonical identity map on ${\textsf{R}}$, and denote $\mathbb {1}_R$ as the identity operator on ${\textsf{R}}$. For any linear map ${\mathscr {N}}_{A\rightarrow B}: {\mathcal {B}}({\textsf{A}}) \rightarrow {\mathcal {B}}({\textsf{B}})$, we define the diamond norm [82, 83] as

2.1 $$\begin{aligned} \left\| {\mathscr {N}}\right\| _{\diamond } \,{:=}\, \sup _{\left\Vert {{\rho _{AR}}}\right\Vert _{1} =1} \left\| {\mathscr {N}}_{A\rightarrow B}\otimes \textrm{id}_R(\rho _{AR}) \right\| _1, \end{aligned}$$

where $${\textsf{R}} \cong {\textsf{A}}$$.

For any $\alpha \in (0,1)\cup (1,\infty )$, we define the order-$\alpha$ sandwiched quantum Rényi divergence $D_\alpha$ [76, 77] for density operator $\rho \in {\mathcal {S}}({\textsf{A}})$ and positive semi-definite $\sigma \in {\mathcal {B}}({\textsf{A}})$ as

2.2 $$\begin{aligned} D_\alpha (\rho \, \Vert \, \sigma )&\,{:=}\,\frac{\alpha }{{\alpha }-1}\log \left\| \sigma ^{\frac{1-\alpha }{2\alpha }}\rho {\sigma }^{\frac{1-\alpha }{2\alpha }}\right\| _{{\alpha }}, \end{aligned}$$

provided that the support of $\rho$ is contained in that of $\sigma$ or $\alpha < 1$ ; otherwise, it is defined to be positive infinity. We define a *generalized sandwiched Rényi information* [78] for a bipartite state $\rho _{AB} \in {\mathcal {S}}({\textsf{A}}\otimes {\textsf{B}})$ and positive semi-definite $\tau _A \in {\mathcal {B}}({\textsf{A}})$ and the usual *sandwiched Rényi information* as

2.3 $$\begin{aligned} I_\alpha (\rho _{AB} \,\Vert \, \tau _A ) :&= \inf _{\sigma _B \in {\mathcal {S}}({\textsf{B}}) } D_\alpha (\rho _{AB} \,\Vert \, \tau _A\otimes \sigma _B); \end{aligned}$$

2.4 $$\begin{aligned} I_\alpha (A: B)_\rho&\,{:=}\, I_\alpha (\rho _{AB} \,\Vert \, \rho _A ). \end{aligned}$$

Moreover, we define the order-$\alpha$ sandwiched Rényi information for a quantum channel (i.e. completely positive and trace-preserving map) ${\mathscr {N}}_{A\rightarrow B}$ as,^9^

2.5 $$\begin{aligned} I_\alpha ({\mathscr {N}}_{A\rightarrow B}) \,{:=}\, \sup _{\psi _{AR}} I_\alpha (B:R)_{({\mathscr {N}}_{A\rightarrow B}\otimes \textrm{id}_R)(\psi ) }. \end{aligned}$$

Here and throughout this paper, $\sup _{\psi _{AR}}$ denotes maximizing over all pure states $\psi _{AR} \in {\mathcal {S}}({\textsf{A}}\otimes {\textsf{R}})$ and ${\textsf{R}} \cong {\textsf{A}}$.

### Remark 1

If $|{\textsf{B}}|<\infty$, then by the compactness of ${\mathcal {S}}({\textsf{B}})$, lower semi-continuity of the sandwiched Rényi divergence in its second argument [104], and the extreme value theorem, the minimum in Eq. (2.3) can be attained. An iterative algorithm with convergence guarantees was provided in [105]. In this paper, although we do not necessary impose the finite-dimensional assumption on the Hilbert space (especially for the output space), the minimum in Eq. (2.3) can also be attained.

As $\alpha \rightarrow 1$, the above quantities converge monotonically to the Umegaki’s *quantum relative entropy* [106, 107, Lemma 3.5], the generalized mutual information, the usual quantum mutual information, and the quantum mutual information of channel, respectively:

2.6 $$\begin{aligned} \lim _{\alpha \rightarrow 1} D_\alpha (\rho \,\Vert \, \sigma )&= D(\rho \,\Vert \, \sigma ) \,{:=}\, {{\,\textrm{Tr}\,}}\left[ \rho (\log \rho - \log \sigma ) \right] , \end{aligned}$$

2.7 $$\begin{aligned} \lim _{\alpha \rightarrow 1} I_\alpha (\rho _{AB} \,\Vert \, \tau _A )&= I(\rho _{AB} \,\Vert \, \tau _A ) \,{:=}\, D(\rho _{AB} \,\Vert \, \tau _A \otimes \rho _B), \end{aligned}$$

2.8 $$\begin{aligned} \lim _{\alpha \rightarrow 1} I_\alpha (A: B)_\rho&= I(A: B)_\rho \,{:=}\, D(\rho _{AB} \,\Vert \, \rho _A \otimes \rho _B), \end{aligned}$$

2.9 $$\begin{aligned} I({\mathscr {N}}_{A\rightarrow B})&\,{:=}\, \sup _{\psi _{RA}} I (B:R)_{({\mathscr {N}}_{A\rightarrow B}\otimes \textrm{id}_R)(\psi )}. \end{aligned}$$

The above information quantities naturally extend to the multipartite setting (where system *A* now has, say $L\in \mathbb {N}$, subsystems). We define a *multipartite sandwiched Rényi information* for a multipartite state $\rho _{A_1 A_2 \ldots A_L E} \in {\mathcal {S}}({\textsf{A}}_1\otimes {\textsf{A}}_2\otimes \cdots \otimes {\textsf{A}}_L \otimes {\textsf{E}})$ and a quantum broadcast channel ${\mathscr {N}}_{A\rightarrow B_1 B_2 \ldots B_L}:{\mathcal {S}}({\textsf{A}}) \rightarrow {\mathcal {S}}({\textsf{B}}_1 \otimes {\textsf{B}}_2 \otimes \cdots \otimes {\textsf{B}}_L)$ as

2.10 $$\begin{aligned} I_\alpha \left( A_1 : A_2 : \cdots : A_L : E\right) _\rho&\,{:=}\, I_\alpha \left( \rho _{A_1 A_2 \ldots A_L E} \, \Vert \otimes _{\ell \in [L]} \rho _{A_{\ell }} \right) ; \end{aligned}$$

2.11 $$\begin{aligned} I_\alpha ( {\mathscr {N}}_{A\rightarrow B_1 B_2 \ldots B_L} )&\,{:=}\, \max _{\psi _{AR}} I_\alpha \left( B_1 : B_2 : \cdots : B_L : R\right) _{ ({\mathscr {N}}\otimes \textrm{id}_R)(\psi )}. \end{aligned}$$

In particular, we term

2.12 $$\begin{aligned} \lim _{\alpha \rightarrow 1} I_\alpha \left( A_1 : A_2 : \cdots : A_L : E\right) _\rho&\equiv I\left( A_1 : \cdots : A_L: E \right) _\rho \nonumber \\&\,{:=}\, D\left( \rho _{A_1 \ldots A_L E} \,\Vert \otimes _{\ell \in [L]} \rho _{A_\ell } \otimes \rho _E \right) , \end{aligned}$$

2.13 $$\begin{aligned} I( {\mathscr {N}}_{A\rightarrow B_1 B_2 \ldots B_L} )&\,{:=}\, \max _{\psi _{AR}} I\left( B_1 : B_2 : \cdots : B_L : R\right) _{ ({\mathscr {N}}\otimes \textrm{id}_R)(\psi )} \end{aligned}$$

as the *multipartite quantum mutual information* [67, 68] $I\left( A_1: \cdots : A_L: E \right) _\rho$ for state $\rho _{A_1 A_2 \ldots A_L E}$ and the *multipartite quantum mutual information* $I( {\mathscr {N}}_{A\rightarrow B_1 B_2 \ldots B_L} )$ of quantum broadcast channel ${\mathscr {N}}_{A\rightarrow B_1 B_2 \ldots B_L}$, respectively.^10^

We further define the *relative entropy variance* [108, 109] $V(\rho \,\Vert \,\sigma )$, *mutual information variance* ${V(A_1: \cdots : A_L: E)_\rho }$, and *channel dispersion* $V({\mathscr {N}}_{A\rightarrow B_1 B_2 \ldots B_L})$ for a broadcast channel ${\mathscr {N}}_{A\rightarrow B_1 B_2 \ldots B_L}$ as

2.14 $$\begin{aligned} V(\rho \,\Vert \,\sigma )&\,{:=}\, {{\,\textrm{Tr}\,}}[\rho (\log \rho - \log \sigma )^2] - \left[ D(\rho \,\Vert \,\sigma ) \right] ^2; \end{aligned}$$

2.15 $$\begin{aligned} V\left( A_1 : \cdots : A_L : E\right) _\rho&\,{:=}\, V\left( \rho _{A_1 \ldots A_L E} \,\Vert \otimes _{\ell \in [L]} \rho _{A_\ell } \otimes \rho _E \right) ; \end{aligned}$$

2.16 $$\begin{aligned} V({\mathscr {N}}_{A\rightarrow B_1 B_2 \ldots B_L})&\,{:=}\, \sup _{\psi _{AR}: I(B_1:\cdots :B_L:R)_{({\mathscr {N}}\otimes \textrm{id}_R)(\psi )} = I({\mathscr {N}}) } V(B_1:\cdots :B_L:R)_{({\mathscr {N}}\otimes \textrm{id}_R)(\psi )}. \end{aligned}$$

Below we collect several well-known properties of the generalized Rényi information. Essentially they all follow similarly as the special case $L=1$ [77, 107, 78, Lemma 7], [23, 110], and [45, Lemma 17].

### Proposition 1

(Properties of Rényi information). For any multipartite state $\rho _{A_1 A_2 \ldots A_L E}$ and $\tau _{A_\ell } \in {\mathcal {S}}({\textsf{A}}_{\ell })$, $\ell \in [L]$, and $\alpha \ge 1/2$, the sandwiched Rényi information satisfies the following properties.

- (Monotone decreasing [77, 107, Lemma 4.6]) When ${\alpha }\rightarrow 1$,
  2.17 $$\begin{aligned} I_\alpha \left( \rho _{A_1 A_2 \ldots A_L E}\, \Vert \otimes _{\ell \in [L]} \tau _{A_\ell } \right) \searrow I\left( \rho _{A_1 A_2 \ldots A_L E}\, \Vert \otimes _{\ell \in [L]} \tau _{A_\ell } \right) . \end{aligned}$$
- (Additivity [78, Lemma 7]) For any integer $n \in \mathbb {N}$,
  2.18 $$\begin{aligned} I_\alpha \left( \rho _{A_1 A_2 \ldots A_L E}^{\otimes n}\, \Vert \otimes _{\ell \in [L]} \tau _{A_\ell }^{\otimes n} \right) = n I_\alpha \left( \rho _{A_1 A_2 \ldots A_L E}\, \Vert \otimes _{\ell \in [L]} \tau _{A_\ell } \right) . \end{aligned}$$
- (Concavity in $\alpha$ [110, Theorem 11])^11^ The following map is concave and upper semi-continuous on $(1,\infty )$:
  2.19 $$\begin{aligned} \alpha \mapsto \frac{1-\alpha }{\alpha } I_\alpha \left( \rho _{A_1 A_2 \ldots A_L E}\, \Vert \otimes _{\ell \in [L]} \tau _{A_\ell } \right) . \end{aligned}$$
- (Concavity in $\psi$ [45, Lemma 17]) The following map is concave and upper semi-continuous on ${\mathcal {S}}({\textsf{A}})$:
  2.20 $$\begin{aligned} \rho _{A} \mapsto I_{\alpha }\left( B_1: B_2: \cdots B_L : R \right) _{{\mathscr {N}}_{A\rightarrow B_1 B_2\ldots B_L}(\psi _{AR}^\rho )}, \end{aligned}$$
  where $\psi _{AR}^\rho$ is a purification of $\rho _A$ and ${\textsf{R}} \cong {\textsf{A}}$.

The following Lemmas 1 and 2 will be used in Sect. 5 for broadcast channel simulation. We delay their proofs to Appendix A.

### Lemma 1

(Dimension bound). For any states $\rho _{ABC} \in {\mathcal {S}}({\textsf{A}} \otimes {\textsf{B}} \otimes {\textsf{C}})$, $\tau _{A} \in {\mathcal {S}}({\textsf{A}})$, and $\alpha >0$, we have

2.21 $$\begin{aligned} I_\alpha \left( \rho _{ABC} \,\Vert \, \tau _A \right) \le I_\alpha \left( \rho _{AB} \,\Vert \, \tau _A \right) + \frac{2\alpha }{\alpha -1} \log |{\textsf{C}}|. \end{aligned}$$

### Lemma 2

(Convexity). Let *L* be any integer and ${\mathcal {I}}$ be any finite set. Let $\rho _{A_1A_2\ldots A_L E} \,{:=}\, \sum _{i\in {\mathcal {I}}} p_i \rho _{A_1A_2\ldots A_L E}^{i}$ and $\tau _{A_\ell } = \sum _{i\in {\mathcal {I}}} p_i \tau _{A_{\ell }}^{i}$, $\ell \in [L]$ be statistical mixtures of states for any $p_i>0$, $\sum _{i\in {\mathcal {I}}} p_i = 1$. Then, the following holds for every $\alpha \ge 1/2$,

2.22 $$\begin{aligned} \begin{aligned} I_\alpha ( \rho _{A_1\ldots A_L E} \,\Vert \otimes _{\ell \in [L]} \tau _{A_\ell } )&\le \sum \nolimits _{i\in {\mathcal {I}} } p_i I_\alpha \left( \rho _{A_1\ldots A_L E}^{i} \,\Vert \otimes _{\ell \in [L]} \tau _{A_\ell }^{i}\right) + L \cdot H(\{p_i\}_{i\in {\mathcal {I}}})\\&\le \sum \nolimits _{i \in {\mathcal {I}} }p_i I_\alpha \left( \rho _{A_1\ldots A_L E}^{i} \,\Vert \otimes _{\ell \in [L]} \tau _{A_\ell }^{i}\right) + L \log |{\mathcal {I}}|. \end{aligned} \end{aligned}$$

Here, $H(\{p_i\}_{i\in {\mathcal {I}}}) \,{:=}\, - \sum _{i\in {\mathcal {I}}} p_i \log p_i$ denotes the Shannon entropy.

We introduce the error-exponent functions as the *Fenchel–Legendre transform* of the above Rényi information quantities, i.e. for any $r>0$,

2.23 $$\begin{aligned} E_r (\rho _{A_1 A_2 \ldots A_L E} \, \Vert \, \otimes _{\ell \in [L]} \tau _{A_\ell })&\,{:=}\, \sup _{\alpha \in [1,2]} \frac{\alpha -1}{\alpha } \left( r - I_\alpha (\rho _{A_1 A_2 \ldots A_L E} \, \Vert \, \otimes _{\ell \in [L]} \tau _{A_\ell }) \right) ; \end{aligned}$$

2.24 $$\begin{aligned} E_r(A_1 : A_2 : \cdots : A_L : E)_\rho&\,{:=}\, E_r (\rho _{A_1 A_2 \ldots A_L E} \, \Vert \otimes _{\ell \in [L]} \rho _{A_\ell }) ; \end{aligned}$$

2.25 $$\begin{aligned} E_r ({\mathscr {N}}_{A\rightarrow B_1 B_2 \ldots B_L})&\,{:=}\, \sup _{\alpha \in [1,2]} \frac{\alpha -1}{\alpha } \left( r - I_\alpha ( {\mathscr {N}}_{A\rightarrow B_1 B_2 \ldots B_L} ) \right) . \end{aligned}$$

We collect the known properties of the error-exponent function [23, 85, 97, 111–113], which are consequences of the properties of the Rényi information given in Proposition 1. and the minimax theorem [114, §36].

### Proposition 2

(Properties of error-exponent function). For any multipartite state $\rho _{A_1 A_2 \ldots A_L B}$ and any quantum broadcast channel ${\mathscr {N}}_{A\rightarrow B_1 B_2 \ldots B_L}$, the error-exponent function satisfies the following properties.

(i) (Positivity) For any $r>0$,
  2.26 $$\begin{aligned} E_r (\rho _{A_1 A_2 \ldots A_L B} \, \Vert \otimes _{\ell \in [L]} \tau _{A_\ell })> 0&\Longleftrightarrow r > I(\rho _{A_1 A_2 \ldots A_L B} \, \Vert \otimes _{\ell \in [L]} \tau _{A_\ell }); \end{aligned}$$
  2.27 $$\begin{aligned} E_r ({\mathscr {N}}_{A\rightarrow B_1 B_2 \ldots B_L})> 0&\Longleftrightarrow r > I( {\mathscr {N}}_{A\rightarrow B_1 B_2 \ldots B_L}). \end{aligned}$$
(ii) (Additivity) For any integer $n \in \mathbb {N}$ and $r>0$,
  2.28 $$\begin{aligned} E_{nr} \left( \rho _{A_1 A_2 \ldots A_L B}^{\otimes n} \, \Vert \otimes _{\ell \in [L]} \tau _{A_\ell }^{\otimes n} \right) = n E_{{r}}\left( \rho _{A_1 A_2 \ldots A_L B} \, \Vert \otimes _{\ell \in [L]} \tau _{A_\ell } \right) . \end{aligned}$$
(iii) (A minimax identity and saddle-point) Provided that the underlying Hilbert spaces are all finite dimensional, for any $r>0$, there exist a saddle-point $(\psi ,\alpha ) \in {\mathcal {S}}({\textsf{A}}\otimes {\textsf{R}}) \times [1,2]$ [114, §36] such that
  2.29 $$\begin{aligned} E_r({\mathscr {N}}_{A\rightarrow B_1 B_2 \ldots B_L})&= \inf _{\psi _{AR}} E_r(B_1: B_2: \cdots B_L: R)_{({\mathscr {N}}\otimes \textrm{id}_R)(\psi )} \end{aligned}$$
  2.30 $$\begin{aligned}&= \frac{\alpha -1}{\alpha } \left( r - I_\alpha (B_1:B_2\cdots B_L: R)_{({\mathscr {N}}\otimes \textrm{id}_R)(\psi )} \right) . \end{aligned}$$
(vi) (Limiting behavior) Provided that the underlying Hilbert spaces are all finite dimensional, then for any sequence $r_n \,{:=}\, I\left( {\mathscr {N}}_{A\rightarrow B_1 B_2\ldots B_L}\right) + a_n$ satisfying $a_n \downarrow 0$, we have
  2.31 $$\begin{aligned} \liminf _{n\rightarrow \infty }\frac{E_{r_n}({\mathscr {N}})}{a_n^2}&\ge \frac{1}{2V({\mathscr {N}}_{A\rightarrow B_1 B_2\ldots B_L})}. \end{aligned}$$

We delay the proof to Appendix A.

### Remark 2

Proposition 2-(iii) indicates that the error exponent of channel $E_r({\mathscr {N}}_{A\rightarrow B_1 B_2 \ldots B_L})$ can be viewed as the error exponent $E_r(B_1: B_2: \cdots B_L: R)_{({\mathscr {N}}\otimes \textrm{id}_R)(\psi )}$ for the output state with respect to the worst-case input $\psi _{AR}$.

## Convex Splitting

In Ref. [72], part of the authors established a one-shot error-exponent bound for *unipartite* convex splitting, i.e. for any density operators $\rho _{AE} \in {\mathcal {S}}(\mathsf {A\otimes E})$ and $\tau _{A} \in {\mathcal {S}}({\textsf{A}})$, and $M\in {\mathbb {N}}$,

3.1 $$\begin{aligned} \frac{1}{2}\left\| \frac{1}{M} \sum \nolimits _{m\in [M]} \rho _{A_m E} \bigotimes \nolimits _{\bar{m}\in [M]\setminus \{m\}} \tau _{A_{\bar{m}}} - \tau _{A}^{\otimes M} \otimes \rho _E \right\| _1&\le 2^{- E_{\log M}(\rho _{AE} \,\Vert \, \tau _A )}, \end{aligned}$$

where $\rho _{A_m E} = \rho _{AE}$ and $\tau _{A_m} = \tau _A$ for all $m\in [M]$, and the error-exponent function $E_{\log M}$ is defined in Eq. (2.23). While this result is a neat application of complex interpolation, a straightforward generalization to the multipartite case does not give the Rényified quantities simultaneously. The key ingredient to bypass this difficulty is a *mean-zero decomposition lemma* that will be introduced later in Lemmas 3 and 5. We remark that a similar idea is independently proposed by Colomer Saus and Winter for deriving multipartite quantum decoupling theorems, termed the *telescoping trick* in their work [80].

### Bi-partite convex splitting

Let $\rho _{ABE}$ be a tripartite density operator in ${\mathcal {S}}(\mathsf {A\otimes B\otimes E})$, and let $\tau _{AB}$ be a bipartite density operator ${\mathcal {S}}(\mathsf {A\otimes B})$. Given integers *M* and *K*, we define the density operator

3.2 $$\begin{aligned} \begin{aligned} \omega _{A_1\ldots A_M B_1\ldots B_KE} \,{:=}\, \frac{1}{MK}\sum _{(m,k)\in [M]\times [K]} \rho _{A_m B_kE}&\otimes \tau _{A_1} \otimes \tau _{A_2} \cdots \otimes \tau _{A_{m-1}} \otimes \tau _{A_{m+1}} \otimes \cdots \otimes \tau _{A_M}\\&\otimes \tau _{B_1} \otimes \tau _{B_2} \cdots \otimes \tau _{B_{k-1}} \otimes \tau _{B_{k+1}} \otimes \cdots \otimes \tau _{B_K}, \end{aligned} \end{aligned}$$

where for each $m\in [M]$ and $k\in [K]$, we have the systems $A_m\cong A$, $B_k\cong B$ and the states $\rho _{A_m B_k E} = \rho _{AB E}$, $\tau _{A_m} = \tau _A$, and $\tau _{B_k} = \tau _B$. We use trace distance as the error criterion for bipartite convex splitting,

3.3 $$\begin{aligned} \begin{aligned} \Delta _{M,K}(\rho _{ABE} \,\Vert \, \tau _{AB})&\,{:=}\, \frac{1}{2}\left\| \omega _{A_1\ldots A_M B_1\ldots B_KE} - \tau _{A}^{\otimes M} \otimes \tau _{B}^{\otimes K}\otimes \rho _E \right\| _1. \end{aligned} \end{aligned}$$

Given $p\ge 1$, recall that the *Kosaki’s weighted* $L_p$*-norm* with respect to a density operator $\sigma$ [115, 116] is defined as,

3.4 $$\begin{aligned} \begin{aligned} \left\| X\right\| _{p,\sigma }&\,{:=}\, \left( {{\,\textrm{Tr}\,}}\left[ \left| \sigma ^{\frac{1}{2p}} X \sigma ^{\frac{1}{2p}} \right| ^p \right] \right) ^{1/p} \end{aligned} \end{aligned}$$

and the associated *noncommutative weighted *$L_p$*-space* is denoted as $L_p(\sigma )= \left\{ X: \Vert X\Vert _{p, \sigma } < \infty \right\}$. For two positive operators *X* and *Y* we introduce the notation of *non-commutative quotient for* *X* *over* *Y* as^12^

3.5 $$\begin{aligned} \frac{X}{Y} \,{:=}\, Y^{-\frac{1}{2}} X Y^{-\frac{1}{2}}. \end{aligned}$$

Our approach (as in the unipartite convex splitting [72]) is to formulate the error $\Delta _{MK}\left( \rho _{ABE} \, \Vert \, \tau _{AB}\right)$ as the weighted $L_p$ norm and estimate it via complex interpolation. We refer to [72, Appendix B] for a minimal introduction of complex interpolation needed for this paper and the readers are referred to [117] for more information on this topic. We start with rewriting the error quantity in Eq. (3.3) using non-commutative $L_p$-norm. Given a density operator $\tau _{AB}=\tau _A\otimes \tau _B$, we define the $\tau$-preserving conditional expectation

3.6 $$\begin{aligned}&\textrm{E}_{A} : {\mathcal {B}}({\textsf{A}})\rightarrow {\mathcal {B}}({\textsf{A}}), \;X_{A} \mapsto {{\,\textrm{Tr}\,}}_{A}\left[ \tau _{A} X_{A} \right] \cdot \mathbb {1}_{A}; \nonumber \\&\textrm{E}_{B} : {\mathcal {B}}({\textsf{B}})\rightarrow {\mathcal {B}}({\textsf{B}}), \;X_{B} \mapsto {{\,\textrm{Tr}\,}}_{B}\left[ \tau _{B} X_{B} \right] \cdot \mathbb {1}_{B}; \nonumber \\&\textrm{E}_{AB}\,{:=}\,\textrm{E}_A\otimes \textrm{E}_B : {\mathcal {B}}(\mathsf {A\otimes B})\rightarrow {\mathcal {B}}(\mathsf {A\otimes B}). \end{aligned}$$

The following map is an key object in the bipartite analysis.

3.7 $$\begin{aligned} \begin{aligned}&T: {\mathcal {B}}(\mathsf {A\otimes B})\rightarrow {\mathcal {B}}(\mathsf {A\otimes B}) \ , \ T\,{:=}\,\textrm{id}_{AB}- \textrm{E}_A-\textrm{E}_B+\textrm{E}_{AB}. \end{aligned} \end{aligned}$$

Here and in the following, $\textrm{E}_A$ can be interpreted as $\textrm{E}_A\otimes \textrm{id}_B$ and similar for $\textrm{E}_B$. We will shortly see how the map *T* comes into play. Let us first state the following lemma that estimates its norm between $L_p$-spaces.

#### Lemma 3

Let $\tau =\tau _{A}\otimes \tau _{B}\otimes \tau _{E} \in {\mathcal {S}}({\textsf{A}}\otimes {\textsf{B}}\otimes {\textsf{E}})$ be a tripartite product density operator and a map *T* introduced in Eq. (3.7). Then

$$\begin{aligned} \left\Vert {{T: L_1(\tau _{ABE})\rightarrow L_1(\tau _{ABE})}}\right\Vert  \le 4\hspace{.1cm}, \hspace{.1cm}\left\Vert {{T: L_2(\tau _{ABE})\rightarrow L_2(\tau _{ABE})}}\right\Vert  \le 2.\end{aligned}$$

#### Proof

We first argue for $L_1$. Note that $\textrm{E}_A \textrm{E}_B= \textrm{E}_B \textrm{E}_A= \textrm{E}_{AB}$. By triangle inequality, it suffices to show both $\textrm{E}_A$ and $\textrm{E}_B$ are contraction. Indeed, consider the duality $L_1(\tau )^*=L_\infty (\tau )$, where the pairing is given the $\tau$-inner product

$$\begin{aligned} \langle X,Y \rangle _\tau ={{\,\textrm{Tr}\,}}\left[ { X^{\dagger } } \tau ^{1/2}Y\tau ^{1/2}\right] \hspace{.1cm}. \end{aligned}$$

Note that $\textrm{E}_A$ is completely positive unital map, hence a contraction on $L_\infty (\tau )\cong {\mathcal {B}}(\mathsf {A\otimes B\otimes E})$ (equipped with usual operator norm). Also, as $\textrm{E}_A$ is self-adjoint for $\tau$-inner product. Then

$$\begin{aligned} \left\Vert {{ \textrm{E}_A:L_1(\tau )\rightarrow L_1(\tau )}}\right\Vert  =\left\Vert {{ \textrm{E}_A\,{:=}\,L_\infty (\tau )\rightarrow L_\infty (\tau )}}\right\Vert  =1. \end{aligned}$$

The same argument applies for $\textrm{E}_B$. Thus we prove that $\left\Vert {{T: L_1\rightarrow L_1}}\right\Vert  \le 4$ by triangle inequality.

For $L_2$, we note that $\textrm{E}_A$ is a $\tau$-preserving conditional expectation. That is, ${{\,\textrm{Tr}\,}}[\tau \textrm{E}_A(X)]={{\,\textrm{Tr}\,}}[\tau X]$ for all $X\in {\mathcal {B}}({\textsf{A}}\otimes {\textsf{B}}\otimes {\textsf{E}})$, and $\textrm{E}_A^2=\textrm{E}_A$ is a unital complete positive idempotent. In particular, $\textrm{E}_A$ is a projection on $L_2(\tau )$. As $\textrm{E}_B$ is a projection commute with $\textrm{E}_A$, we have

$$\begin{aligned} X-\textrm{E}_A(X)-\textrm{E}_B(X)+\textrm{E}_{AB}(X)=(X-\textrm{E}_A(X))-\textrm{E}_B(X-\textrm{E}_{A}(X)) . \end{aligned}$$

Hence

$$\begin{aligned} \left\Vert {{X}}\right\Vert _{L_2(\tau )}\ge \left\Vert {{X-\textrm{E}_A(X)}}\right\Vert _{L_2(\tau )}\ge \left\Vert {{\textrm{E}_B(X)-\textrm{E}_{AB}(X)}}\right\Vert _{L_2(\tau )}. \end{aligned}$$

The second assertion follows from triangle inequality for $L_2(\tau )$. $\square$

In the following, we use the short notation $A^M\,{:=}\,A_1\cdots A_M\cong A^{\otimes M}$ and $B^K\,{:=}\,B_1\cdots B_K\cong B^{\otimes K}$. Given $(m,k)\in [M]\times [K]$, we define the following maps

$$\begin{aligned} \pi _{m,k}:&\;{\mathcal {B}}({\textsf{A}} \otimes {\textsf{B}})\rightarrow {\mathcal {B}}({\textsf{A}}^{M} \otimes {\textsf{B}}^{K})\\&\;\;X_{AB} \mapsto \mathbb {1}_{A_1 B_1}\otimes \cdots \otimes \mathbb {1}_{A_{m-1} B_{k-1}} \otimes X_{A_m B_k} \otimes \mathbb {1}_{A_{m+1} B_{k+1}} \otimes \cdots \otimes \mathbb {1}_{A_M B_K};\\ \textrm{E}_{A_m} :&\;X_{A_m} \mapsto {{\,\textrm{Tr}\,}}_{A_m}\left[ \rho _{A_m} X_{A_m} \right] \cdot \mathbb {1}_{A_m};\\ \textrm{E}_{B_k} :&\;X_{B_k} \mapsto {{\,\textrm{Tr}\,}}_{B_k}\left[ \rho _{B_k} X_{B_k} \right] \cdot \mathbb {1}_{B_k};\\ \Theta \,{:=}\,&\;\frac{1}{MK}\sum _{(m,k)\in [M]\times [K]} \pi _{m,k} \end{aligned}$$

Here, the condition expectation $\textrm{E}_{A_m}$ is only acting on the ${A}_m$ system while other systems remain unchanged; similarly for $\textrm{E}_{B_k}$. It is clear that

$$\begin{aligned} \pi _{m,k}\textrm{E}_A=\textrm{E}_{A_m}\pi _{m,k}\, \pi _{m,k}\textrm{E}_B=\textrm{E}_{B_k}\pi _{m,k}\, \pi _{m,k}\textrm{E}_{AB}=\textrm{E}_{A_mB_k}\pi _{m,k} \hspace{.1cm}. \end{aligned}$$

Take $\tau _{ABE}=\tau _A\otimes \tau _B\otimes \sigma _E$ for an arbitrary density $\sigma _E\in {\mathcal {S}}({\textsf{E}})$. We have

3.8 $$\begin{aligned} \Delta _{M,K}\left( \rho _{ABE}\,\Vert \,\tau _{AB} \right)&= \frac{1}{2}\left\| \omega _{A_1\ldots A_M B_1\ldots B_KE} - \tau _{A}^{\otimes M} \otimes \tau _{B}^{\otimes K}\otimes \rho _E \right\| _1 \end{aligned}$$

3.9 $$\begin{aligned}&= \frac{1}{2}\left\| \frac{\omega _{A_1\ldots A_M B_1\ldots B_KE}}{\tau _{A^MB^KE}} - \mathbb {1}_{A^MB^K}\otimes \frac{\rho _E}{\sigma _E} \right\| _{L_1(\tau _{A^MB^KE})} \end{aligned}$$

3.10 $$\begin{aligned}&= \frac{1}{2}\left\| \frac{1}{MK}\sum _{m,k}\pi _{m,k}\left( \frac{\rho _{ABE}}{\tau _A\otimes \tau _B\otimes \sigma _E}\right) -\mathbb {1}_{A^MB^K}\otimes \frac{\rho _E}{\sigma _E} \right\| _{L_1(\tau _{A^MB^KE})} \end{aligned}$$

3.11 $$\begin{aligned}&= \frac{1}{2}\left\| \frac{1}{MK}\sum _{m,k}\pi _{m,k}\left( \frac{\rho _{ABE}}{\tau _A\otimes \tau _B\otimes \sigma _E}-\mathbb {1}_{AB}\otimes \frac{\rho _E}{\sigma _E}\right) \right\| _{L_1(\tau _{A^MB^KE})} \end{aligned}$$

3.12 $$\begin{aligned}&= \frac{1}{2}\left\| \frac{1}{MK}\sum _{m,k}\pi _{m,k}\circ (\textrm{id}_{AB}-\textrm{E}_{AB}) \left( \frac{\rho _{ABE}}{\tau _A\otimes \tau _B\otimes \sigma _E}\right) \right\| _{L_1(\tau _{A^MB^KE})} \end{aligned}$$

3.13 $$\begin{aligned}&= \frac{1}{2}\left\| \Theta \circ (\textrm{id}_{AB}-\textrm{E}_{AB}) \left( \frac{\rho _{ABE}}{\tau _A\otimes \tau _B\otimes \sigma _E}\right) \right\| _{L_1(\tau _{A^MB^KE})}. \end{aligned}$$

A key observation is that, for $X\equiv X_{ABE}=\frac{\rho _{ABE}}{\tau _A\otimes \tau _B\otimes \sigma _E}$, we can decompose in Eq. (3.13) into the following three terms:

3.14 $$\begin{aligned}&(\textrm{id}_{AB}-\textrm{E}_{AB})(X)=X-\textrm{E}_{AB}(X)=(X-\textrm{E}_AX-\textrm{E}_BX+\textrm{E}_{AB}X)\nonumber \\&+(\textrm{E}_AX-\textrm{E}_{AB}X)+(\textrm{E}_BX-\textrm{E}_{AB}X)\hspace{.1cm}. \end{aligned}$$

Let us evaluate one of them:

3.15 $$\begin{aligned}&\left\| \Theta (\textrm{E}_AX-\textrm{E}_{AB}X) \right\| _{L_1(\tau _{A^MB^KE})}\nonumber \\&\quad =\left\Vert {{\Theta \left( \mathbb {1}_{A}\otimes \frac{\rho _{BE}}{\tau _B\otimes \sigma _E}-\mathbb {1}_{AB}\otimes \frac{\rho _{E}}{ \sigma _E}\right) }}\right\Vert _{L_1(\tau _{A^MB^KE})}\nonumber \\&\quad = \left\Vert {{ \frac{1}{MK}\sum _{m,k} \mathbb {1}_{A^M}\otimes \frac{\rho _{B_kE}}{\tau _B\otimes \sigma _E}\otimes \mathbb {1}_{B_1\cdots B_{k-1}B_{k+1}\cdots B_K} - { \mathbb {1}_{A^M B^K} } \otimes \frac{\rho _E}{\sigma _E}}}\right\Vert _{L_1(\tau _{A^MB^KE})}\nonumber \\&\quad =\left\Vert {{ \frac{1}{K}\sum _{k} \tau _{A^M}\otimes \rho _{B_kE}\otimes \tau _{B_1} \otimes \tau _{B_2} \cdots \otimes \tau _{B_{k-1}} \otimes \tau _{B_{k+1}} \otimes \cdots \otimes \tau _{B_K} - \tau _{A^MB^K} \otimes \rho _E}}\right\Vert _{1}\nonumber \\&\quad =\left\Vert {{ \tau _{A^M}}}\right\Vert _{1}\cdot \left\Vert {{ \frac{1}{K}\sum _{k} \rho _{B_kE}\otimes \tau _{B_1} \otimes \tau _{B_2} \cdots \otimes \tau _{B_{k-1}} \otimes \tau _{B_{k+1}} \otimes \cdots \otimes \tau _{B_K} - \tau _{B^K} \otimes \rho _E}}\right\Vert _{1}\nonumber \\&\quad = 2\Delta _{K}\left( \rho _{BE} \,\Vert \, \tau _B\right) . \end{aligned}$$

which is exactly the unipartite convex-splitting error for density operators $\rho _{BE}$ and $\tau _B$. Similarly,

$$\begin{aligned} \left\| \Theta (\textrm{E}_BX-\textrm{E}_{AB}X) \right\| _{L_1(\tau _{A^MB^KE})}&= { 2 } \Delta _{K}\left( \rho _{AE} \, \Vert \, \tau _A\right) . \end{aligned}$$

Given the unipartite convex-split result in Eq. (3.1), it suffices to deal with the term

$$\begin{aligned} \Theta (X-\textrm{E}_AX-\textrm{E}_BX+\textrm{E}_{AB}X)=\Theta \circ T(X) \hspace{.1cm}. \end{aligned}$$

Using Lemma 3 for estimating the norm of map *T*, we obtain a key technical lemma to bound the norm $\Theta \circ T$ as follows.

#### Lemma 4

(Map norm of bipartite biconvex splitting). Given any $M,K\in \mathbb {N}$ and density operator $\tau _{ABE}=\tau _A\otimes \tau _B\otimes \sigma _E$, let $\Theta$ and *T* be the map defined as in Eq. (3.6). Then, for every density operator $\sigma _E$, we have

3.16 $$\begin{aligned} \left\| \Theta \circ T : L_{p}(\tau _{ABE}) \rightarrow L_{p}(\tau _{A^MB^KE}) \right\| \le 2^{\frac{3}{p}-1} (MK)^{ \frac{1-p}{p} }, \quad \forall \, p\in [1,2]. \end{aligned}$$

#### Proof

We first note that for any $p\in [1,\infty ]$ and every $(m,k)\in [M]\times [K]$,

3.17 $$\begin{aligned} \pi _{m,k}: L_{p}(\tau _{ABE}) \rightarrow L_{p}(\tau _{A^MB^KE}) \end{aligned}$$

is an isometry. Indeed,

$$\begin{aligned} \left\Vert {{\pi _{m,k}(X_{ABE})}}\right\Vert _{L_{p}(\tau _{A^MB^KE})}&=\left\Vert {{\mathbb {1}_{A^{M/\{m\} }B^{K/\{k\}}}\otimes X_{A_kB_mE}}}\right\Vert _{L_{p}(\tau _{A^MB^KE})}\\&=\left\Vert {{ \tau _{A^{M/\{m\} }B^{K/\{k\}}}^{\frac{1}{p}}\otimes \tau _{A_mB_k}^{\frac{1}{2p}}X_{A_kB_mE}\tau _{A_mB_k}^{\frac{1}{2p}}}}\right\Vert _{p}\\&=\left\Vert {{ \tau _{ABE}^{\frac{1}{2p}}X_{ABE}\tau _{ABE}^{\frac{1}{2p}}}}\right\Vert _{p}=\left\Vert {{X_{ABE}}}\right\Vert _{L_{p}(\tau _{ABE})} \end{aligned}$$

For $p=1$, by triangle inequality we have

3.18 $$\begin{aligned}&\left\| \Theta : L_{1}(\tau _{ABE}) \rightarrow L_{1}(\tau _{A^MB^KE}) \right\| \le \frac{1}{MK}\sum _{m,k} \Vert \pi _{m,k}: L_{1}(\tau _{ABE}) \rightarrow L_{1}(\tau _{A^MB^KE}) \Vert \le 1, \end{aligned}$$

and hence by Lemma 3

$$\begin{aligned} \left\| \Theta \circ T: L_{1}(\tau _{ABE}) \rightarrow L_{1}(\tau _{A^MB^KE}) \right\| \le 4\hspace{.1cm}. \end{aligned}$$

For $p=2$, we note that for every $(m,k)\ne (m',k')\in [M]\times [K]$, the range of $\pi _{m,k}\circ T$ and $\pi _{m',k'}\circ T$ are mutually orthogonal in $L_2( \tau _{A^MB^KE})$. Indeed, without loss of generality, we assume $k\ne k'$, and for any $X_{ABE},Y_{ABE}$ denote $\mathring{X}\,{:=}\,T(X)=X-\textrm{E}_AX-\textrm{E}_BX+\textrm{E}_{AB}X$. Then

3.19 $$\begin{aligned}&\langle \pi _{m,k}(\mathring{X}), \pi _{m',k'}(\mathring{Y}) \rangle _{\tau _{A^MB^KE}} \end{aligned}$$

3.20 $$\begin{aligned}&\quad = \langle \mathbb {1}_{A^{[M]/\{m\}}B^{[K]/{k}}} \otimes \mathring{X}_{A_m B_kE}, \mathbb {1}_{A^{[M]/\{m'\}}B^{[K]/{k'}}} \otimes \mathring{X}_{A_{m'} B_{k'}E}\rangle _{\tau _{A^MB^KE}} \end{aligned}$$

3.21 $$\begin{aligned}&\quad = {{\,\textrm{Tr}\,}}\Big (\tau _{A^MB^KE}^{\frac{1}{2}}\mathring{X}_{A_m B_kE} \tau _{A^MB^KE}^{\frac{1}{2}}\mathring{Y}_{A_{m'} B_{k'}E}\Big ) \end{aligned}$$

3.22 $$\begin{aligned}&\quad = {{\,\textrm{Tr}\,}}\Big (\tau _{A_mA_{m'}B_kB_{k'}E}^{\frac{1}{2}}\mathring{X}_{A_m B_kE} \tau _{A_mA_{m'}B_kB_{k'}E}^{\frac{1}{2}}\mathring{Y}_{A_{m'} B_{k'}E}\Big ) \end{aligned}$$

3.23 $$\begin{aligned}&\quad = {{\,\textrm{Tr}\,}}\Big (\tau _{A_mA_{m'}E}^{\frac{1}{2}}\textrm{E}_{B_{k}}(\mathring{X}_{A_mB_kE}) \tau _{A_mA_{m'}E}^{\frac{1}{2}}\textrm{E}_{B_{k'}}(\mathring{X}_{A_{m'}B_{k'} E})\Big ) \end{aligned}$$

3.24 $$\begin{aligned}&\quad = {{\,\textrm{Tr}\,}}\Big (\tau _{A_mA_{m'}E}^{\frac{1}{2}}(\pi _{m,k}\circ \textrm{E}_B(\mathring{X})) \tau _{A_mA_{m'}E}^{\frac{1}{2}}(\pi _{m',k'}\circ \textrm{E}_B(\mathring{Y}))\Big ) \end{aligned}$$

3.25 $$\begin{aligned}&\quad = 0 \end{aligned}$$

because $\textrm{E}_B\mathring{X}=\textrm{E}_B\mathring{Y}=0$. In the second last inequality, we used the commutation relation $\textrm{E}_{B_{k}}\pi _{m,k}=\pi _{m,k}\textrm{E}_B$.

By the orthogonality, for any $X_{ABE}$

3.26 $$\begin{aligned} \left\| \frac{1}{MK} \sum _{m,k} \pi _{m,k} \left( \mathring{X} \right) \right\| _{L_2(\tau _{A^MB^KE})}^2&\overset{\text {(a)}}{=} \frac{1}{M^2 K^2} \sum _{m,k} \left\| \pi _{m,k} \left( \mathring{X} \right) \right\| _{L_2(\tau _{A^MB^KE})}^2 \end{aligned}$$

3.27 $$\begin{aligned}&\overset{\text {(b)}}{=} \frac{1}{M^2 K^2} \sum _{m,k} \left\| \mathring{X} \right\| _{L_2(\tau _{ABE})}^2 \end{aligned}$$

3.28 $$\begin{aligned}&= \frac{1}{MK} \left\| \mathring{X} \right\| _{L_2(\tau _{ABE})}^2 \end{aligned}$$

3.29 $$\begin{aligned}&\overset{\text {(c)}}{\le } \frac{4}{MK} \left\| X \right\| _{L_2(\tau _{ABE})}^2 \end{aligned}$$

where (a) follows from orthogonality; (b) is because $\pi _{m,k}$ are isometry; and the last inequality (c) follows by the Lemma 3. The case of general $p\in [1,2]$ follows from complex interpolation for $\frac{1}{p}=\frac{1-\theta }{1}+\frac{\theta }{2}$, $\theta = \frac{2(p-1)}{p} \in [0,1]$,

3.30 $$\begin{aligned} \left\| \Theta \circ T: L_{p} \rightarrow L_{p} \right\|&\le \left\| \Theta \circ T : L_{1} \rightarrow L_{1}\right\| ^{1-\theta } \left\| \Theta \circ T : L_{2} \rightarrow L_{2}\right\| ^\theta = 2^{\frac{3-p}{p} }(MK)^{ \frac{1-p}{p} }, \end{aligned}$$

which completes the proof. $\square$

#### Remark 3

The above estimate holds for asymmetric case $L_{p,\gamma }(\tau )$ with norm $\left\Vert {{X}}\right\Vert _{L_p(p,\gamma ,\tau )}=\left\| \tau ^{\frac{1-\gamma }{p}} X\tau ^{\frac{\gamma }{p}} \right\| _{p}$ for any $\gamma \in [0,1]$, although this point will not be used in our discussion.

Combining with unipartite convex splitting $\Delta _M(\rho _{AE}\Vert \tau _A),\Delta _K(\rho _{BE}\Vert \tau _B)$ given in Eq. (3.1), we have

#### Theorem 1

(2-party convex splitting) For any density operators $\rho _{ABE} \in {\mathcal {S}}(\mathsf {A\otimes B\otimes E})$ and $\tau _{AB} \in {\mathcal {S}}(\mathsf {A\otimes B})$, and $M,K\in {\mathbb {N}}$, we have

3.31 $$\begin{aligned} \begin{aligned} \Delta _{M,K}(\rho _{ABE} \,\Vert \, \tau _{AB})&\le 2\cdot \,2^{ - E_{\log MK}(\rho _{ABE} \,\Vert \, \tau _A \otimes \tau _B ) } + 2^{- E_{\log M}(\rho _{AE} \,\Vert \, \tau _A )} + 2^{- E_{\log K}(\rho _{BE} \,\Vert \, \tau _B )}, \end{aligned} \end{aligned}$$

where the error exponents are defined in Eq. (2.23).

Moreover, the exponents are all negative if and only if,

3.32 $$\begin{aligned} {\left\{ \begin{array}{ll} & \log MK> I(\rho _{ABE} \,\Vert \, \tau _A \otimes \tau _B ), \\ & \log M> I(\rho _{AE} \,\Vert \, \tau _A ),\\ & \log K > I(\rho _{BE} \,\Vert \, \tau _B ).\\ \end{array}\right. } \end{aligned}$$

#### Remark 4

Theorem 1 holds for infinite-dimensional Hilbert spaces ${\textsf{A}}$, ${\textsf{B}}$, and ${\textsf{E}}$ as well.

#### Proof

The proof is a combination of the analysis before. By (3.13), the bipartite convex splitting error can be estimated as

3.33 $$\begin{aligned} \Delta _{M,K}\left( \rho _{ABE}\,\Vert \,\tau _{AB} \right)&\,{:=}\, \frac{1}{2}\left\| \omega _{A_1\ldots A_M B_1\ldots B_KE} - \tau _{A}^{\otimes M} \otimes \tau _{B}^{\otimes K}\otimes \rho _E \right\| _1 \end{aligned}$$

3.34 $$\begin{aligned}&= \frac{1}{2}\left\| \Theta \circ (\textrm{id}_{AB}-\textrm{E}_{AB}) \left( \frac{\rho _{ABE}}{\tau _A\otimes \tau _B\otimes \sigma _E}\right) \right\| _{L_1(\tau _{A^MB^KE})}. \end{aligned}$$

According to the mean zero decomposition for $X_{ABE}=\frac{\rho _{ABE}}{\tau _A\otimes \tau _B\otimes \sigma _E}$,

3.35 $$\begin{aligned} (\textrm{id}_{AB}-\textrm{E}_{AB})(X)&=(X-\textrm{E}_AX-\textrm{E}_BX+\textrm{E}_{AB}X)+(\textrm{E}_AX-\textrm{E}_{AB}X)+(\textrm{E}_BX-\textrm{E}_{AB}X)\hspace{.1cm}\end{aligned}$$

3.36 $$\begin{aligned}&=T(X)+(\textrm{E}_AX-\textrm{E}_{AB}X)+(\textrm{E}_BX-\textrm{E}_{AB}X)\hspace{.1cm}, \end{aligned}$$

we have by the triangle inequality,

3.37 $$\begin{aligned} \Delta _{M,K}\left( \rho _{ABE}\,\Vert \,\tau _{AB} \right) \le \frac{1}{2}\left\| \Theta (T(X))\right\| _{L_1(\tau _{A^MB^KE})}&+ \frac{1}{2}\left\| \Theta (\textrm{E}_AX-\textrm{E}_{AB}X)\right\| _{L_1(\tau _{A^MB^KE})} \end{aligned}$$

3.38 $$\begin{aligned}&+\frac{1}{2}\left\| \Theta (\textrm{E}_BX-\textrm{E}_{AB}X)\right\| _{L_1(\tau _{A^MB^KE})}. \end{aligned}$$

Here, the map *T* was defined in (3.7).

By the calculation (3.15) and unipartite convex splitting estimate (3.1), the second and third terms are unipartite convex splitting error

3.39 $$\begin{aligned}&\frac{1}{2}\left\| \Theta (\textrm{E}_AX-\textrm{E}_{AB}X)\right\| _{L_1(\tau _{A^MB^KE})}=\Delta _{K}\left( \rho _{BE}\,\Vert \,\tau _{B} \right) \le 2^{- E_{\log K}(\rho _{BE} \,\Vert \, \tau _B )}, \end{aligned}$$

3.40 $$\begin{aligned}&\frac{1}{2}\left\| \Theta (\textrm{E}_BX-\textrm{E}_{AB}X)\right\| _{L_1(\tau _{A^MB^KE})}=\Delta _{K}\left( \rho _{AE}\,\Vert \,\tau _{A} \right) \le 2^{- E_{\log M}(\rho _{AE} \,\Vert \, \tau _A )}\hspace{.1cm}. \end{aligned}$$

For the first term, we apply the map norm of $\Theta \circ T$ from Lemma 4, then for any $p\in [1,2]$ and density operator $\sigma _E$,

$$\begin{aligned} \frac{1}{2}\left\| \Theta \circ T(X)\right\| _{L_1(\tau _{A^MB^KE})}&\le \frac{1}{2}\left\| \Theta \circ T(X)\right\| _{L_p(\tau _{A^MB^KE})}\\&\le \frac{1}{2}\left\| \Theta \circ T : L_{p}(\tau _{ABE}) \rightarrow L_{p}(\tau _{A^MB^KE}) \right\| \left\Vert {{X}}\right\Vert _{L_p(\tau _{A^MB^KE})}\\&\le \frac{1}{2}\cdot 2^{\frac{3}{p}-1} (MK)^{ \frac{1-p}{p} } \left\Vert {{\frac{\rho _{ABE}}{\tau _A\otimes \tau _B\otimes \sigma _E}}}\right\Vert _{L_p(\tau _{A^MB^KE})}\\&= 2^{\frac{3}{p}-2} 2^{-\frac{p-1}{p}(\log MK ) } 2^{\frac{p-1}{p} D_p(\rho _{ABE}||\tau _A\otimes \tau _B\otimes \sigma _E) }\\&\le 2\cdot 2^{-\frac{p-1}{p}(\log MK -D_p(\rho _{ABE}||\tau _A\otimes \tau _B\otimes \sigma _E)) }. \end{aligned}$$

Here, the first inequality is consequence of Hölder’s inequality that for any density operator $\tau$, $1\le p\le \infty$ and $\frac{1}{p}+\frac{1}{p'}=1$,

$$\begin{aligned}\left\| Y\right\| _{L_1(\tau )}=\left\| \tau ^{\frac{1}{2}}Y \tau ^{\frac{1}{2}}\right\| _1\le \left\| \tau ^{\frac{1}{2p'}}\right\| _{2p'}\left\| \tau ^{\frac{1}{2p}}Y \tau ^{\frac{1}{2p}}\right\| _p \left\| \tau ^{\frac{1}{2p'}}\right\| _{2p'}=\left\| Y\right\| _{L_p(\tau )}\hspace{.1cm}.\end{aligned}$$

Taking infimum over all $p\in [1,2]$ and density operator $\sigma$ yields

3.41 $$\begin{aligned} \frac{1}{2}\left\| \Theta \circ T(X)\right\| _{L_1(\tau _{A^MB^KE})}\le 2\cdot \,2^{ - E_{\log MK}(\rho _{ABE} \,\Vert \, \tau _A \otimes \tau _B ) } \end{aligned}$$

Combining the estimates (3.39), (3.40) and (3.41) for the three parts, we have the desired inequality

3.42 $$\begin{aligned} \Delta _{M,K}(\rho _{ABE} \,\Vert \, \tau _{AB})&\le 2\cdot \,2^{ - E_{\log MK}(\rho _{ABE} \,\Vert \, \tau _A \otimes \tau _B ) } + 2^{- E_{\log M}(\rho _{AE} \,\Vert \, \tau _A )} + 2^{- E_{\log K}(\rho _{BE} \,\Vert \, \tau _B )}. \end{aligned}$$

$$\square $$

### Multipartite convex splitting

In this section, we derive multipartite convex splitting. Following the “pedestrian” argument for the bipartite case, we will focus more on illustrating the mathematical structure of convex splitting. Let $A_1,\cdots , A_L$ be *L* systems. For each $\ell \in [L]$, we denote $A_\ell ^{M_{\ell }}\,{:=}\,A_{\ell ,1}\cdots A_{\ell ,M_\ell }\cong A_\ell ^{\otimes M_{\ell }}$ as $M_\ell$ copies of system $A_\ell$. Given a multipartite state $\rho _{A_1 \ldots A_L E}$, a product state $\tau _{A_1 \ldots A_L}=\tau _{A_1}\otimes \cdots \otimes \tau _{A_L}$, and integers $M_1$, $M_2$, $\ldots$, $M_L$, we define

3.43 $$\begin{aligned} \omega _{A^{M_1} \cdots A^{M_L} E} \,{:=}\, \frac{1}{M_1 \cdots M_L}\sum _{m_\ell \in [M_\ell ], \, \ell \in [L]} \rho _{A_{ 1, m_1} \cdots A_{ L, m_L} E} \left( \bigotimes _{ \bar{m}_{\ell } \in [M_{\ell }]/\{m_\ell \}, \, \ell \in [L] } \tau _{ A_{\ell , \bar{m}_{\ell } } },\right) \end{aligned}$$

where for each $\ell \in [L]$, $m_\ell ,\bar{m}_\ell \in [M_L]$, we let $\rho _{A_{ 1, m_1} \cdots A_{ L, m_L} E} = \rho _{A_1 \ldots A_L E}$ and $\tau _{A_{\ell , \bar{m}_{\ell } }} = \tau _{A_\ell }$. The error for the *L*-partite convex splitting is

3.44 $$\begin{aligned} \begin{aligned} \Delta _{M_1, \cdots , M_L}\left( \rho _{A_1 \ldots A_L E} \,\Vert \, \tau _{A_1 \ldots A_L} \right)&\,{:=}\, \frac{1}{2}\left\| \,\omega _{A^{M_1} \cdots A^{M_L} E} - \bigotimes _{\ell \in [L]}\tau _{A_\ell }^{\otimes M_\ell } \otimes \rho _{E}\, \right\| _1. \end{aligned} \end{aligned}$$

The key argument in the multipartite case is the decomposition map as we introduced in Eq. (3.7) for the bipartite case. For a set $S=\{j_1,\cdots , j_k\}\subseteq [L]$, we introduce the following notation:

$$\begin{aligned}&\rho _{A_{S} E}=\rho _{A_{j_{1}}\ldots A_{j_k} E}\hspace{.1cm}, \; \tau _{A_{S}}=\tau _{A_{j_{1}}}\otimes \cdots \otimes \tau _{A_{j_k} }\hspace{.1cm}, \\ \hspace{.1cm}&M_{S}=\prod \nolimits _{\ell \in {S}}M_{j}\hspace{.1cm}. \end{aligned}$$

Given the product density operator $\tau _{A_{[L]}}=\tau _{A_1}\otimes \cdots \otimes \tau _{A_{L}}$, we define the $\tau$-preserving conditional expectation:

$$\begin{aligned}&\textrm{E}_{A_\ell }: {\mathcal {B}}(A_\ell ) \rightarrow {\mathcal {B}}(A_\ell )\hspace{.1cm}, \hspace{.1cm}\textrm{E}_{A_\ell }(X)\,{:=}\,{{\,\textrm{Tr}\,}}[X\tau _{A_{\ell }}] \mathbb {1}_{A_{\ell }} \hspace{.1cm},\\&\textrm{E}_{{S}}\,{:=}\,\prod \nolimits _{\ell \in {S}} \textrm{E}_{A_{\ell }} : {\mathcal {B}}(A_{S}) \rightarrow {\mathcal {B}}(A_{S})\hspace{.1cm}.\\&\overset{\circ }{\textrm{E}}_{A_\ell }\,{:=}\,\textrm{id}_{A_\ell }-\textrm{E}_{A_\ell }\\&\overset{\circ }{\textrm{E}}_{{S}}\,{:=}\,\prod \nolimits _{\ell \in {S}} \overset{\circ }{\textrm{E}}_{A_{\ell }} : {\mathcal {B}}(A_{S}) \rightarrow {\mathcal {B}}(A_{S})\hspace{.1cm}.\\ \end{aligned}$$

Here and in the following, the map $\textrm{E}_{R}\,{:=}\,\textrm{E}_{R}\otimes \textrm{id}_{R^c}$ are also interpreted as $\textrm{E}_R= \prod _{\ell \in R} \textrm{E}_{A_{\ell }}$ on $A_R$ and identity map on other systems $A_{R^c}$ (similar for $\textrm{E}_{A_{\ell }}$). The order of the product here does not matter because the conditional expectations $\textrm{E}_{A_{\ell }}$ (resp. $\overset{\circ }{\textrm{E}}_{A_\ell }$) are commuting projections on $L_2(\tau )$ onto the subspace of operators trivial on $A_\ell$ (resp. mean zero on $A_\ell$). For a subset $S\subseteq [L]$ and its complement $S^c=[L]{\setminus } {S}$, we define the *mean-zero map*:

3.45 $$\begin{aligned}&T_{{S}}: {\mathcal {B}}(A_{[L]}) \rightarrow {\mathcal {B}}(A_{[L]})\hspace{.1cm}, \hspace{.1cm}\end{aligned}$$

3.46 $$\begin{aligned}&T_{{S}}\,{:=}\,\textrm{E}_{{S}^c} \overset{\circ }{\textrm{E}}_{{S}} \end{aligned}$$

3.47 $$\begin{aligned}&\quad \; =\sum \nolimits _{{S}^c\subseteq R \subseteq [L]} (-1)^{|R\setminus {S}^c|} \textrm{E}_{ R } \end{aligned}$$

The key property of the mean-zero map is that $T_{S}$ projects a multi-partite operator *X* into its “*S*-mean-zero” part $T_{S}(X)$, which i) supported on $A_S$ (identity on other systems) and ii) all partial condition expectations (e.g. a weighted partial trace) from *S* are zero. We remark that a similar idea is independently proposed by Colomer Saus and Winter for deriving multipartite quantum decoupling theorems, termed the *telescoping trick* in their work [80].

#### Lemma 5

(Mean-zero decomposition). Let ${S}\subseteq [L]$ be a subset and $\ell \in [L]$. Then

(i) $\textrm{E}_\ell T_{S}=T_S$ if $\ell \notin S$ and $\textrm{E}_\ell T_{S}=0$ if $\ell \in S$.
(ii) We have
  3.48 $$\begin{aligned} \textrm{E}_{{S}^c} X= \sum \nolimits _{R\subseteq {S} } T_{R} ( X). \end{aligned}$$
  In particular,
  3.49 $$\begin{aligned} X-\textrm{E}_{[L]} X= \sum \nolimits _{\varnothing \ne {S}\subseteq [L] }T_{{S}}(X). \end{aligned}$$

#### Proof

By the definition of $T_S$, if $\ell \in S^c$, $\textrm{E}_\ell \textrm{E}_{{S}^c}=\textrm{E}_{{S}^c}$,

$$\begin{aligned} \textrm{E}_\ell T_S= \textrm{E}_\ell \textrm{E}_{{S}^c} \overset{\circ }{\textrm{E}}_{{S}}=\textrm{E}_{{S}^c}\overset{\circ }{\textrm{E}}_{{S}}=T_S. \end{aligned}$$

If $\ell \in S$, since $\textrm{E}_\ell \circ {\textrm{E}}_{A_{\ell }}=0$ and all these condition expectations are commuting maps, i.e.,

$$\begin{aligned}&\textrm{E}_\ell \overset{\circ }{\textrm{E}}_{{S}}= \textrm{E}_\ell \prod \nolimits _{\ell '\in {S}} \overset{\circ }{\textrm{E}}_{A_{\ell '}}=\textrm{E}_\ell \overset{\circ }{\textrm{E}}_{A_{\ell }} \prod \nolimits _{\ell '\in {S},\ell '\ne \ell } \overset{\circ }{\textrm{E}}_{A_{\ell '}}=0;\\&\textrm{E}_\ell T_{{S}}= \textrm{E}_\ell \overset{\circ }{\textrm{E}}_{{S}}\textrm{E}_{{S}^c}=0. \end{aligned}$$

The first assertion is a direct application of Fubini’s theorem, i.e.

$$\begin{aligned} \textrm{E}_{{S}^c}&=\textrm{E}_{{S}^c}\textrm{id}_{A_S} = \textrm{E}_{{S}^c}\prod _{\ell \in S } \textrm{id}_{A_\ell } = \textrm{E}_{{S}^c}\prod _{\ell \in S } (E_{A_\ell }+\textrm{id}_{A_\ell }-E_{A_\ell }) = \textrm{E}_{{S}^c}\prod _{\ell \in S } (E_{A_\ell }+\overset{\circ }{\textrm{E}}_{A_\ell }) \\  &= \textrm{E}_{{S}^c}\sum _{R\subset {S}} \left( \prod _{\ell \in R } \overset{\circ }{\textrm{E}}_{A_\ell }\prod _{\ell \in S \backslash R } E_{A_\ell }\right) \\&=\sum _{R\subset {S}} \left( \prod _{\ell \in R } \overset{\circ }{\textrm{E}}_{A_\ell }\prod _{\ell \in R^c } E_{A_\ell }\right) \\&= \sum _{R\subset {S}} \overset{\circ }{\textrm{E}}_{R} \textrm{E}_{{R}^c} \\  &= \sum _{R\subset {S}} T_{R} \end{aligned}$$

The second assertion follows from $T_{\varnothing }=E_{[L]}$ and

$$\begin{aligned} X-\textrm{E}_{[L]} X=\textrm{E}_{\varnothing } X-\textrm{E}_{[L]} X= \sum _{{S}\subset [L]} T_{{S}} ( X) -T_{\varnothing } (X)= \sum _{\varnothing \ne {S}\subset [L] }T_{{S}}(X). \end{aligned}$$

$$\square $$

By triangle inequality, the norm of the mean-zero map can be estimate as follows.

#### Lemma 6

For any ${S}\subset [L]$ and $p\in [1,\infty ]$

$$\begin{aligned}\left\Vert {{T_{S}:L_p(\tau _{A_{[L]}}\otimes \sigma _E) \rightarrow L_p(\tau _{A_{[L]}}\otimes \sigma _E)}}\right\Vert  \le 2^{|{S}|} \hspace{.1cm}. \end{aligned}$$

#### Proof

It suffices to note that for each $R\subset [L]$, the $\tau$-preserving conditional expectation $\textrm{E}_R$ is a contraction on $L_p(\tau )$ for all $p\in [1,\infty ]$. $\square$

#### Remark 5

The $L_2$ estimate for $T_{S}$ can be at least improved to

$$\begin{aligned} \left\Vert {{T_{S}:L_2(\tau _{A_{[L]}}\otimes \sigma _E) \rightarrow L_2(\tau _{A_{[L]}}\otimes \sigma _E)}}\right\Vert  \le 2^{|{S}|-1}.\end{aligned}$$

We now discussing the multipartite convex splitting map. Given a multi-index $\textbf{m}=(m_1,\cdots , m_\ell )\in [M_1]\times \cdots [M_L]$, we introduce the short notation

3.50 $$\begin{aligned}&A_{\textbf{m}}=A_{1,m_1}\cdots A_{L,m_L}\hspace{.1cm}, \hspace{.1cm}A_{\textbf{m}^c}=\bigotimes _{ \bar{m}_\ell \in [M_{\ell }]\setminus \{m_{\ell }\}, \ell \in [L] }A_{\ell ,\bar{m}_\ell }\hspace{.1cm}, \end{aligned}$$

3.51 $$\begin{aligned}&A_\textbf{m}\otimes A_{\textbf{m}^c}= \bigotimes _{m_\ell \in [M_\ell ],\ell \in [L]} A_{\ell ,m_\ell }\cong A_1^{\otimes M_1}\cdots A_L^{\otimes M_L}.\end{aligned}$$

Define the following map

$$\begin{aligned} \pi _{\textbf{m}}:&\;{\mathcal {B}}(\mathsf {A_1\cdots A_L E})\rightarrow {\mathcal {B}}(\mathsf {A_1^{M_1}\cdots A_L^{M_L}E})\ \\&\; X_{A_1\cdots A_L E} \mapsto X_{A_{\textbf{m}} E} \otimes \mathbb {1}_{A_{\textbf{m}}^c}; \\ \textrm{E}_{A_{\ell , m_\ell }} :&\;{\mathcal {B}}(A_{\ell , m_\ell })\rightarrow {\mathcal {B}}(A_{\ell , m_\ell })\hspace{.1cm}\\&\;X \mapsto {{\,\textrm{Tr}\,}}\left[ \tau _{A_\ell } X \right] \cdot \mathbb {1}_{A_{\ell ,m_\ell }};\\ \Theta _{[L]}\,{:=}\,&\;\frac{1}{M_{[L]}}\sum _{\textbf{m}\in [M_1]\times \cdots \times [M_L]} \pi _{\textbf{m}}. \end{aligned}$$

#### Lemma 7

For any $\textbf{m}$, $\pi _{\textbf{m}}$ is an isometry from $L_p(\tau _{A_1\cdots A_L}\otimes \sigma _E)$ to $L_p\left( \tau _{A_1^{M_1}\cdots A_L^{M_L}}{\otimes } \sigma _E\right)$ for all $p\in [1,\infty ]$. Thus, by triangle inequality,

#### Proof

The proof is similar to the bipartite case in Lemma 4$\square$

Similar to the bipartite case, the condition expectation $\textrm{E}_{A_{\ell , m_\ell }}$ is only acting on the ${A}_{\ell , m_\ell }$ system andwhere $A_{S,\textbf{m}_S}=\prod _{\ell \in S} A_{\ell , m_\ell }$. Given the density $\tau _{A_1\cdots A_L}=\tau _{A_1}\otimes \cdots \otimes \tau _{A_L}$, we writeFor an arbitrary full rank density $\sigma _E\in S({\textsf{E}})$, the multipartite convex splitting error can be expressed by

$$\begin{aligned}&\Delta _{M_1, \cdots , M_L}\left( \rho _{A_1 \ldots A_L E} \,\Vert \, \tau _{A_1 \ldots A_L} \right) \\&\quad =\frac{1}{2}\left\Vert {{\frac{1}{M_{[L]}}\sum _{\textbf{m}} \rho _{A_{\textbf{m}} E}\otimes \tau _{A_{\textbf{m}}^c}-\bigotimes _{\ell \in [L]}\tau _{A_\ell }^{\otimes M_\ell } \otimes \rho _{E} }}\right\Vert _{1}\\&\quad =\frac{1}{2}\left\Vert {{\frac{1}{M_{[L]}}\sum _{\textbf{m}} \frac{\rho _{A_{\textbf{m}} E}\otimes \tau _{A_{\textbf{m}}^c}}{\tau _{A_1^{M_1}\cdots A_L^{M_L}}\otimes \sigma _E}-\bigotimes _{\ell \in [L]}{\mathbb {1}}_{A_\ell }^{\otimes M_\ell } \otimes \frac{\rho _{E}}{\sigma _E} }}\right\Vert _{L_1(\tau _\textbf{A}\otimes \sigma _E)} \\ \quad&=\frac{1}{2}\left\Vert {{\frac{1}{M_{[L]}}\sum _{\textbf{m}} \frac{\rho _{A_{\textbf{m}} E}}{\tau _{A_{\textbf{m}}}\otimes \sigma _E} \otimes \textbf{1}_{A_{\textbf{m}}^c}-\bigotimes _{\ell \in [L]}{\mathbb {1}}_{A_\ell }^{\otimes M_\ell } \otimes \frac{\rho _{E}}{\sigma _E} }}\right\Vert _{L_1(\tau _\textbf{A}\otimes \sigma _E)} \\ \quad&=\frac{1}{2}\left\Vert {{\frac{1}{M_{[L]}}\sum _{\textbf{m}}\pi _{\textbf{m}} \left( \frac{\rho _{A_{ 1} \cdots A_{ L} E}}{\tau _{A_1 \ldots A_L}\otimes \sigma _E} -{\mathbb {1}}_{A_1\cdots A_L}\otimes \frac{\rho _{E}}{\sigma _E} \right) }}\right\Vert _{L_1(\tau _\textbf{A}\otimes \sigma _E)} \\ \quad&=\frac{1}{2}\left\Vert {{\Theta _{[L]} \left( \textrm{id}_{[L]}-\textrm{E}_{[L]}\right) (X)}}\right\Vert _{L_1(\tau _\textbf{A}\otimes \sigma _E)}. \end{aligned}$$

where $X=\frac{\rho _{A_{ 1} \cdots A_{ L} E}}{\tau _{A_1 \ldots A_L}\otimes \sigma _E}$. Using the mean-zero decomposition lemma given in Lemma 5,3.52where for each subset $S\subseteq [L]$, we define the error termNote that $T_S(X)=\textbf{1}_{A_{S^c} }\otimes \mathring{X}_S$ is supported on $A_{S}$ where $\mathring{X}_S=T_S(X_S)$, so the convex splitting for $A_{S^c}$ is trivial. More precisely, one havewhich is an |*S*|-partite convex splitting term $\Theta _{[S]}\circ T_{[S]}$. Here, $\textbf{A}_{S}=\bigotimes _{m_\ell \in [M_\ell ], \ell \in S} A_\ell$, and we use the notation ${\textbf{m}_S}= (m_\ell )_{\ell \in S}$ as the restriction of multi-index $\textbf{m}\in \times _{\ell \in [L]} [M_\ell ]$ to $\textbf{m}\in \times _{\ell \in S} [M_\ell ]$.

#### Lemma 8

For any subset $S\subseteq [L]$ and any density operator $\sigma _E$,As a consequence,

#### Proof

By the above observation, it suffices to argue for the case $S=[L]$. Recall that by Hölder inequality, for a density operator $\tau$, the identity map $\textrm{id}: L_p(\tau )\rightarrow L_1(\tau )$ is a contraction. Then it suffices to show that for $p\in [1,2]$

$$\begin{aligned}\left\Vert {{\Theta _{[L]}\circ T_{[L]}:L_p(\tau _{A_1\cdots {A_L}}\otimes \sigma _E)\rightarrow L_p\left( \tau _{A_1^{M_1}\cdots A_L^{M_L}}\otimes \sigma _E\right) }}\right\Vert  \le 2^{|S|}M_{[L]}^{\frac{1-p}{p}}\hspace{.1cm}.\end{aligned}$$

For $p=1$, we use Lemmas 6 and 7

$$\begin{aligned} \left\Vert {{\Theta _{[L]}\circ T_{[L]}:L_1\rightarrow L_1 }}\right\Vert  \le \left\Vert {{\Theta _{[L]}:L_1\rightarrow L_1 }}\right\Vert  \left\Vert {{ T_{[L]}:L_1\rightarrow L_1 }}\right\Vert  =2^{L}\hspace{.1cm}. \end{aligned}$$

For $p=2$, given an operator $X\in {\mathcal {B}}({\textsf{A}}_1\otimes \ldots \otimes {\textsf{A}}_L\otimes E)$, we adopt the short notation $\mathring{X}=T_{[L]}(X)$. Note that by Lemma 5, $\textrm{E}_{A_\ell } (\mathring{X})=0$ for all $\ell \in [L]$. This implies that the set $\{\pi _{\textbf{m}}(\mathring{X} )\}_{\textbf{m}\in [M_1]\times \cdots \times [M_L]}$ is orthogonal in $L_2(\tau _\textbf{A}\otimes \sigma _E)$. Indeed, for $\textbf{m}\ne \textbf{m}'$, without loss of generality, assume $m_{1}\ne m'_1$. Write $\tau _{\textbf{A}E}=\tau _\textbf{A} \otimes \sigma _E$. We have

$$\begin{aligned}&\langle \pi _{\textbf{m}}(\mathring{X}), \pi _{\textbf{m}'}(\mathring{Y}) \rangle _{\tau _{\textbf{A}E}} \\&\quad = \langle \mathbb {1}_{A_{\textbf{m}^c}E} \otimes \mathring{X}_{A_\textbf{m}}, \mathbb {1}_{A_{{\textbf{m}'}^c}} \otimes \mathring{Y}_{A_{\textbf{m}'}E}\rangle _{\tau _{\textbf{A}E}}\\&\quad = {{\,\textrm{Tr}\,}}\left[ \tau _{\textbf{A}E}^{\frac{1}{2}}\mathring{X}_{A_\textbf{m}} \tau _{\textbf{A}E}^{\frac{1}{2}}\mathring{Y}_{A_{\textbf{m}'}E}\right] \\&\quad = {{\,\textrm{Tr}\,}}\left[ \tau _{A_{\textbf{m}\cup \textbf{m}'}E}^{\frac{1}{2}}\mathring{X}_{A_\textbf{m}} \tau _{A_{\textbf{m}\cup \textbf{m}'}E}^{\frac{1}{2}}\mathring{Y}_{A_{\textbf{m}'}E}\right] \\&\quad = {{\,\textrm{Tr}\,}}\left[ \tau _{A_{\textbf{m}\cup \textbf{m}'\setminus \{m_1,m_1'\} } E}^{\frac{1}{2}}E_{A_{1,m_1}}(\mathring{X}_{A_\textbf{m}}) \tau _{A_{\textbf{m}\cup \textbf{m}'\setminus \{m_1,m_1'\}}E}^{\frac{1}{2}}E_{A_{1,m_1'}}(\mathring{Y}_{A_{\textbf{m}'}E})\right] \\&\quad = \langle E_{A_{1,m_1}}(\pi _{\textbf{m}}(\mathring{X})), E_{A_{1,m_1'}}(\pi _{\textbf{m}'}(\mathring{Y})) \rangle _{ \tau _{\textbf{A}E} }\\&\quad = \langle \pi _{\textbf{m}}\circ E_{A_1}( \mathring{X}), \pi _{\textbf{m}'}\circ E_{A_1}(\mathring{Y}) \rangle _{ \tau _{\textbf{A}E} }\\&\quad = 0. \end{aligned}$$

By the orthogonality, for any $X_{A_1\cdots A_L E}$

3.53 $$\begin{aligned} \left\| \Theta _{[L]}(\mathring{X}) \right\| _{L_2(\tau _{\textbf{A}E})}^2&\overset{\text {(a)}}{=} \frac{1}{M_{[L]}^2} \sum _{\textbf{m}} \left\| \pi _{\textbf{m}} \left( \mathring{X} \right) \right\| _{L_2(\tau _{\textbf{A}E})}^2 \nonumber \\&\overset{\text {(b)}}{=} \frac{1}{M_{[L]}^2} \sum _{\textbf{m}} \left\| \mathring{X} \right\| _{L_2(\tau _{A_1\cdots A_L E})}^2 \nonumber \\&= \frac{1}{M_{[L]}} \left\| \mathring{X} \right\| _{L_2(\tau _{A_1\cdots A_L E})}^2 \nonumber \\&\overset{\text {(c)}}{\le } \frac{2^{2L}}{M_{[L]}} \left\| X \right\| _{L_2(\tau _{A_1\cdots A_L E})}^2 \end{aligned}$$

where (a) follows from orthogonality; (b) is because $\pi _{\textbf{m}}$ are isometry; and the last inequality (c) follows by the Lemma 6. For general $p\in [1,2]$, we apply complex interpolation for $\frac{1}{p}=\frac{1-\theta }{1}+\frac{\theta }{2}$, $\theta = \frac{2(p-1)}{p} \in [0,1]$,

$$\begin{aligned}&\left\| \Theta _{[L]}\circ T_{[L]}: L_{p} \rightarrow L_{p} \right\|&\le \left\| \Theta _{[L]}\circ T_{[L]}: L_{1} \rightarrow L_{1}\right\| ^{1-\theta } \left\| \Theta _{[L]}\circ T_{[L]}: L_{2} \rightarrow L_{2}\right\| ^\theta \\&= 2^L(M_{[L]})^{ \frac{1-p}{p} }, \end{aligned}$$

which finishes the proof for $S=[L]$. The consequence follows from taking infimum over all $\sigma _E$ and $p\in (1,2]$. $\square$

#### Remark 6

As we mentioned, the key property of the element $\mathring{X}\,{:=}\,T_{[L]}(X)$ is that $\mathring{X}$ has all “partial mean” zero $E_\ell T_S(X)=0, \forall \ell \in [L]$. Indeed, for each $\ell \in [L]$, the partial mean-zero $E_\ell (\mathring{X})=0$ implies that for any fixed $(m_1,\cdots , m_{\ell -1}, m_{\ell +1},m_{L} )$ the set $\{\pi _\textbf{m}( \mathring{X})\}_{m_{\ell }=1}^{M_\ell }$ is orthogonal. Then to make the whole set $\{\pi _\textbf{m}( \mathring{X})\}_{\textbf{m}\in \prod [M_\ell ]}$ orthogonal is exactly our motivation for the mean-zero decomposition Lemma 5.

Using triangle inequality, we have the following one-shot multipartite convex splitting lemma. Note that in applying the above estimate the density operator $\sigma _E$ can be optimized differently for each term $\Delta _S$.

#### Theorem 2

(*L*-party convex splitting). Let $\rho _{A_1A_2\ldots A_L E}$ and $\tau _{A_1 \ldots A_L}=\tau _{A_1}\otimes \tau _{A_2} \cdots \otimes \tau _{A_L}$ be multipartite states. For integers $M_1$, $M_2$, $\ldots$, $M_L \in \mathbb {N}$,

3.54 $$\begin{aligned} \Delta _{M_1, \cdots , M_L}\left( \rho _{A_1 \ldots A_L E} \,\Vert \, \tau _{A_1 \ldots A_L} \right) \le \frac{1}{2}\sum _{\varnothing \ne S\subseteq [L] } 2^{|S|} \cdot 2^{ -E_{\log M_S}\left( \rho _{A_S E} \,\Vert \, \tau _{A_S}\right) }, \end{aligned}$$

where the error-exponent function is defined in Eq. (2.23), $M_S\,{:=}\, \Pi _{ \ell \in S } M_\ell$, and $A_S$ denotes systems $A_\ell$ for all $\ell \in S$. Moreover, the error exponents are all positive if and only if for all subsets $S\subseteq [L]$,

3.55 $$\begin{aligned} \sum \nolimits _{\ell \in S} \log M_\ell > I_1(\rho _{A_SE} \,\Vert \, \tau _{A_S} ). \end{aligned}$$

#### Proof

This is a consequence of (3.52) and Lemma 8,

$$\begin{aligned} \Delta _{M_1, \cdots , M_L}\left( \rho _{A_1 \ldots A_L E} \,\Vert \, \tau _{A_1 \ldots A_L} \right)&\le \sum _{\varnothing \ne S\subseteq [L]}\Delta _S(\rho _{A_1\cdots A_LE} \,\Vert \, \tau _{A_1\cdots A_{L}})\hspace{.1cm},\\&\le \sum _{\varnothing \ne S\subseteq [L]}2^{|S|-1} \cdot 2^{-E_{\log M_S}( \rho _{A_SE}||\tau _{A_S} )}\hspace{.1cm}. \end{aligned}$$

$$\square $$

#### Remark 7

Theorem 2 holds even if the underlying Hilbert spaces are all infinite-dimensional.

## Multipartite Quantum State Splitting

**Fig. 3:**
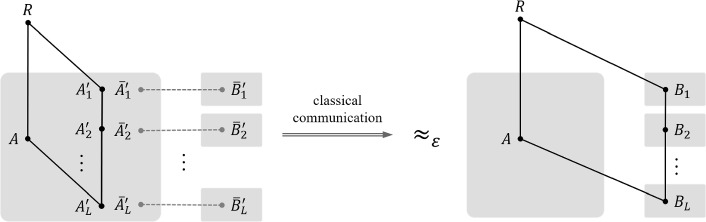
Depiction of *L*-party Quantum State Splitting protocol. Sender holds registers *A*, $A_1'$, $\bar{A}_1'$, $A_2'$, $\bar{A}_2'$, $\cdots$, $A_L'$, $\bar{A}_L'$, and pre-shares entanglement with each of the *L* Receivers holding register $B_\ell '$. $\ell \in [L]$; register *R* represents an inaccessible Reference. Each grey-shaded region is allowed to perform local quantum operation at its holding registers, and (limited) noiseless classical communication from the sender to the *L* receivers is permitted. After executing the protocol, we wish the systems $A_1'$, $\cdots$, $A_L'$ end up at each of the $\ell$-th receiver’s side (at the right part of the figure). Note that each gray-shaded region is only allowed to perform local quantum operation

In this section, we derive the one-shot multipartite Quantum State Splitting. We first provide a formal definition (see Fig. 3).

### Definition 1

(*L-party Quantum State Splitting*)

Let $\rho _{RAA_1'A_2'\ldots A_L'}$ be a pure state as input of the protocol.

1. Quantum registers $A_1'$, $A_2'$, $\ldots$, $A_L'$ and *A* at Alice, $B_1 \cong A_1'$, $B_2 \cong A_2'$, $\ldots$, $B_L \cong A_L'$ at *L* Receivers, and *R* at an inaccessible Reference.
2. A resource of entanglement, say $|\tau \rangle _{\bar{A}_1' \bar{A}_2' \ldots \bar{A}_L \bar{B}_1' \bar{B}_2' \ldots \bar{B}_L'}$, shared between Sender (holding registers $\bar{A}_1' \bar{A}_2' \ldots \bar{A}_L'$) and each of the *L* Receivers (each holding register $\bar{B}_\ell '$, $\ell \in [L]$), and noiseless one-way classical communication from the sender to *L* receivers are available. Note that, entanglement between receivers is not needed.
3. The sender applies a local operation on her system and the shared entanglement to obtain *L*-tuple $\vec {r} \,{:=}\, (r_1, r_2, \ldots r_L)$ bits of classical messages.
4. The sender sends the above message to each receiver, respectively, via noiseless one-way classical communication.
5. Upon receiving the messages, each receiver applies a local operation on his shared entanglement to obtain an overall state ${\widehat{\rho }}_{RAB_1 B_2 \ldots B_L}$.

A $( \vec {r}, \varepsilon )$ *L*-party Quantum State Splitting protocol for $\rho _{RAA_1'\ldots A_L'}$ with entanglement $|\tau \rangle _{\bar{A}_1' \bar{A}_2' \ldots \bar{A}_L \bar{B}_1' \bar{B}_2' \ldots \bar{B}_L'}$ satisfies

4.1 $$\begin{aligned} \frac{1}{2}\left\| {\widehat{\rho }}_{RAB_1 \ldots B_L} - \rho _{RAB_1\ldots B_L} \right\| _1 \le \varepsilon , \end{aligned}$$

where $$\rho _{RAB_1 \ldots B_L} \,{:=}\, (\textrm{id}_{A_1'\ldots A_L' \rightarrow B_1 \ldots B_L} \otimes \textrm{id}_{AR}) \rho _{RAA_1'A_2'\ldots A_L'}$$.

For readability, we first show a special case of the bipartite Quantum State Splitting.

### Theorem 3

(2-party Quantum State Splitting). For any pure state $\rho _{AA_1'A_2'R} = |\rho \rangle \langle \rho |_{AA_1'A_2'R}$, there exists a $(r_1, r_2, \varepsilon )$ 2-party Quantum State Splitting protocol for $\rho _{RAA_1'A_2'}$ with entanglement $|\tau \rangle _{{A}_1' {B}_1}$ and $|\tau \rangle _{{A}_2' {B}_2}$ such that

4.2 $$\begin{aligned} \varepsilon&\le \sqrt{ 2 \cdot 2^{ - E_{r_1+r_2} \left( \rho _{A_1'A_2'R} \, \Vert \, \tau _{A_1'} \otimes \tau _{A_2'} \right) } + 2^{ - E_{r_1} \left( \rho _{A_1'R} \, \Vert \, \tau _{A_1'} \right) } + 2^{ - E_{r_2} \left( \rho _{A_2'R} \, \Vert \, \tau _{A_2'} \right) } }, \end{aligned}$$

where the error-exponent functions are defined in Eq. (2.23). Moreover, the error exponents are all positive if and only if,

4.3 $$\begin{aligned} {\left\{ \begin{array}{ll} & r_1+r_2> I(\rho _{A_1'A_2'R} \,\Vert \, \tau _{A_1'} \otimes \tau _{A_2'} ), \\ & r_1> I(\rho _{A_1'R} \,\Vert \, \tau _{A_1'} ),\\ & r_2 > I(\rho _{A_2'R} \,\Vert \, \tau _{A_2'} ).\\ \end{array}\right. } \end{aligned}$$

### Proof

Unipartite Quantum State Splitting has been shown via unipartite convex splitting in Ref. [66] (see also [66]), Below we will demonstrate applying their approach with the newly established bipartite convex splitting (Theorem 1) to achieving a bipartite Quantum State Splitting protocol using $r_1$ and $r_2$ bits of classical communication with the desired error $\varepsilon$.

Let $M = 2^{r_1}$ and $K = 2^{r_2}$. We fix two states $\tau _{A_1'}$ and $\tau _{A_2'}$ that will be specify later. To begin the protocol, we let the sender (Alice) and the first receiver (Bob 1) share *M*-copies of entanglement $\otimes _{m\in [M]} |\tau \rangle _{A_{1,m}' B_{1,m}}$, and Alice and the second receiver (Bob 2) share *K*-copies of entanglement $\otimes _{k\in [K]} |\tau \rangle _{A_{2,k}' B_{2,k}}$, where for each $(m,k) \in [M]\times [K]$, Bob 1 holds register $B_{1,m} \cong B_1$ that purifies Alice’s register $A_{1,m}' \cong A_1'$, and Bob 2 holds register $B_{2,k} \cong B_2$ that purifies register Alice’s $A_{2,k}' \cong A_2'$. Hence, we start with the following pure state:

4.4 $$\begin{aligned} |\overline{\omega }\rangle \,{:=}\, |\rho \rangle _{A A_{1}' A_{2}' R } \otimes _{m\in [M]} |\tau \rangle _{A_{1,m}' B_{1,m}} \otimes _{k\in [K]} |\tau \rangle _{A_{2,k}' B_{2,k}}. \end{aligned}$$

Suppose, hopefully, by the protocol, we end up with the following pure state:

4.5 $$\begin{aligned} |{\omega }\rangle&\,{:=}\, \frac{1}{\sqrt{MK}} \sum _{(m,k)\in [M] \times [K] } |m\rangle _M |k\rangle _K |\rho \rangle _{A B_{1,m} B_{2,k} R } |0\rangle _{A_{1,m}' A_{2,k}'}\nonumber \\&\otimes _{\bar{m} \in [M]\setminus \{m\}} |\tau \rangle _{A_{1,\bar{m}}' B_{1,\bar{m}}} \otimes _{\bar{k} \in [K]\setminus \{k\}} |\tau \rangle _{A_{2,\bar{k}}' B_{2,\bar{k}}}, \end{aligned}$$

where Alice holds registers *M*, *K*, *A*, $A_{1,[M]}' \,{:=}\, A_{1,1}'\ldots A_{1,M}'$ and $A_{2,[K]}' \,{:=}\, A_{2,1}' \ldots A_{2,K}'$, and Bob 1 and Bob 2 hold registers $B_{1,[M]} \,{:=}\, B_{1,1} \ldots B_{1,M}$ and $B_{2,[K]} \,{:=}\, B_{2,1} \cdots B_{2,K}$, respectively. Here, we shorthand for $A_{1,[M]}' \,{:=}\, A_{1,1}'\ldots A_{1,M}'$ and $A_{2,[K]}' \,{:=}\, A_{2,1}' \ldots A_{2,K}'$ (and similarly for $B_{1,[M]}$ and $B_{2,[K]}$) to ease the burden of notation. (We slightly abuse notation to use *M* and *K* denoting registers representing classical systems [*M*] and [*K*].) Alice measures on registers *M* and *K*, and send on the outcome *m* to Bob 1 via $r_1$ bits of classical communication, and the outcome *k* to Bob 2 via $r_2$ bits of classical communication. Then, the two receivers can pick up registers $B_{1,m}$ and $B_{2,k}$, respectively, to end up with $|\rho \rangle _{A B_{1,m} B_{2,k} R } \cong |\rho \rangle _{A B_1 B_2 R }$, which is exactly the target state we aimed for the bipartite Quantum State Splitting protocol.

In what follows, we will show that there exists a local operation protocol at Alice such that we can approximate $|{\omega }\rangle$ in Eq. (4.5) within an error $\varepsilon$ no larger than the right-hand side of Eq. (4.2). Note that a reduced state of $|\overline{\omega }\rangle$ is

4.6 $$\begin{aligned} \overline{\omega }_{A_{1,[M]}'A_{2,[K]}'R}&= \otimes _{m\in [M]} \tau _{A_{1,m}'} \otimes _{k\in [K]} \tau _{A_{2,k}'} \otimes \rho _R. \end{aligned}$$

Then, the bipartite convex split lemma (Theorem 1) guarantees that $\overline{\omega }_{A_{1,[M]}'A_{2,[K]}'R}$ can be approximated by a state

4.7 $$\begin{aligned} \begin{aligned} \omega _{A_{1,[M]}'A_{2,[K]}' R}&\,{:=}\, \frac{1}{MK} \sum _{(m,k)\in [M] \times [K] } \rho _{A_{1,m}' A_{2,k}' R } \otimes _{\bar{m} \in [M]\setminus \{m\}} \tau _{A_{1,\bar{m}}'} \otimes _{\bar{k} \in [K]\setminus \{k\}} \tau _{A_{2,\bar{k}}'}, \end{aligned} \end{aligned}$$

within an error $\varepsilon '$ (in terms of trace distance) no larger than the right-hand side of Eq. (3.31) (by substituting register *A* by $A_1'$, *B* by $A_2'$, and *E* by *R*). Observe that $\omega _{A_{1,[M]}'A_{2,[K]}' R}$ is a reduced state of the desired pure state $|{\omega }\rangle$ given in Eq. (4.5). Hence, by Uhlmann’s theorem (Fact 1), there exists an isometry *V* acting on register $AA_1'A_2'A_{1,[M]}'A_{2,[K]}'$ to register $MKAA_{1,[M]}'A_{2,[K]}'$ such that $V |\overline{\omega }\rangle$ is $\sqrt{2\varepsilon '}$-close (in trace distance) to $|\omega \rangle$. Moreover, since the isometry *V* only acts on Alice’s registers, this constitutes the bipartite Quantum State Splitting protocol with error $\varepsilon$ no larger than $\sqrt{2\varepsilon '}$. $\square$

The above bipartite Quantum State Splitting is straightforwardly genearlized to any *L*-party case as follows.

### Theorem 4

(*L*-party Quantum State Splitting). For any pure state $\rho _{AA_1'A_2'\ldots A_L' R} = |\rho \rangle \langle \rho |_{AA_1'A_2'\ldots A_L' R}$, there exists a $(\vec {r}, \varepsilon )$ *L*-party Quantum State Splitting protocol for $\rho _{RAA_1'A_2'\ldots A_L' R}$ with entanglement $|\tau \rangle _{{A}_\ell ' {B}_\ell }$, $\ell \in [L]$ such that

4.8 $$\begin{aligned} \varepsilon&\le \sqrt{ \sum \nolimits _{\varnothing \ne S\subseteq [L] } 2^{|S|} \cdot 2^{ - E_{r_S}\left( \rho _{A_S' R} \,\Vert \, \tau _{A_S'}\right) } }, \end{aligned}$$

where the error-exponent function is defined in Eq. (2.23), $r_S\,{:=}\, \sum _{ \ell \in S } r_\ell$, $A_S'$ denotes systems $A_\ell '$ for all $\ell \in S$, and $\tau _{A_S'} \,{:=}\, \otimes _{\ell \in S} \tau _{A_\ell '}$. Moreover, the error exponents are all positive if and only if for all $\varnothing \ne S \subseteq [L]$,

4.9 $$\begin{aligned} r_S> I\left( \rho _{A_S' R} \,\Vert \, \tau _{A_S'}\right) . \end{aligned}$$

## Entanglement-Assisted Quantum Broadcast Channel Simulation

Let ${\mathscr {N}}_{A\rightarrow B_1 B_2\cdots B_L}$ be an *L*-receiver quantum broadcast channel, which is a completely positive and trace-preserving map from system *A* to *L* systems $B_1 B_2 \ldots B_L$.

### Definition 2

*(L-party Quantum Broadcast Channel Simulation).* Let ${\mathscr {N}}_{A\rightarrow B_1 B_2\cdots B_L}$ be a quantum broadcast channel as input of the simulation protocol.

1. Sender holds registers *A*. Each Receiver holds register $B_1$, $B_2$, $\ldots$, $B_L$, respectively.
2. Free resource of perfect entanglement is shared between Sender (holding registers $\bar{A}_1' \bar{A}_2' \ldots \bar{A}_L'$) and each of the *L* Receivers (holding register $\bar{B}_\ell '$, $\ell \in [L]$).
3. Sender applies a local operation on her systems and sends an *L*-tuple $\vec {r} \,{:=}\, (r_1, r_2, \ldots r_L)$ bits of classical information to each of the *L* Receivers, respectively.
4. Upon receiving the message, each Receiver applies a local operation on his own system.

A $( \vec {r}, \varepsilon )$ *L*-party Quantum Broadcast Channel Simulation protocol for ${\mathscr {N}}_{A\rightarrow B_1 B_2\cdots B_L}$ satisfies

5.1 $$\begin{aligned} \frac{1}{2}\left\| \widehat{{\mathscr {N}}}_{A\rightarrow B_1 B_2\cdots B_L} -{\mathscr {N}}_{A\rightarrow B_1 B_2\cdots B_L} \right\| _{\diamond } \le \varepsilon , \end{aligned}$$

where $\widehat{{\mathscr {N}}}_{A\rightarrow B_1 B_2\cdots B_L}$ is the effectively resulting linear transformation from Sender’s register *A* to *L* Receivers’ registers $B_1$, $\ldots$, $B_L$. The *L*-tuple $\vec {r}$ denotes the classical communication costs.

### Definition 3

*(Capacity region for i.i.d. broadcast channel simulation).* The capacity region of simulating ${\mathscr {N}}_{A\rightarrow B_1 B_2\cdots B_L}$, denoted as ${\mathcal {C}}({\mathscr {N}}_{A\rightarrow B_1 B_2\cdots B_L})$ is the closure of

5.2 $$\begin{aligned} \bigcap _{\varepsilon >0} \left\{ \vec {r} \in \mathbb {R}^L: \exists \, (n \vec {r}, \varepsilon )\hbox {-} L \hbox {-party Quantum Broadcast Channel Simulation protocol for} {\mathscr {N}}^{\otimes n} \right\} . \end{aligned}$$

### Theorem 5

(Non-asymptotic achievability for 2-party Quantum Broadcast Channel Simulation). Let ${\mathscr {N}}_{A\rightarrow B_1 B_2}$ be an *L*-receiver quantum broadcast channel. For any integer *n*, there exists an $(n r_1, n r_2, \varepsilon )$ Quantum Broadcast Channel Simulation protocol for ${\mathscr {N}}_{A\rightarrow B_1 B_2}^{\otimes n}$ satisfying

5.3 $$\begin{aligned} \varepsilon&\le k_n \cdot \sqrt{ 2 \cdot \delta _{1,2} + \delta _1 + \delta _2 }, \end{aligned}$$

5.4 $$\begin{aligned} \delta _{1,2}&\,{:=}\, 2^{- n E_{r_1+r_2}({\mathscr {N}}_{A\rightarrow B_1 B_2}) }, \end{aligned}$$

5.5 $$\begin{aligned} \delta _1&\,{:=}\, 2^{- n E_{r_1}({\mathscr {N}}_{A\rightarrow B_1} ) }, \end{aligned}$$

5.6 $$\begin{aligned} \delta _2&\,{:=}\, 2^{- n E_{r_2}({\mathscr {N}}_{A\rightarrow B_2}) }, \end{aligned}$$

where the error-exponent functions are defined in Eq. (2.25), and the polynomial prefactor is $k_n \,{:=}\, (n+1)^{\frac{5}{2} (|{\textsf{A}}|^2-1) }$.

Moreover, the three exponents $E_{r_1+r_2}({\mathscr {N}}_{A\rightarrow B_1 B_2})$, $E_{r_1}({\mathscr {N}}_{A\rightarrow B_1})$, $E_{r_2}({\mathscr {N}}_{A\rightarrow B_2})$ are all positive if and only if

5.7 $$\begin{aligned} {\left\{ \begin{array}{ll} &  r_1 + r_2> I( {\mathscr {N}}_{A\rightarrow B_1 B_2} ), \\ &  r_1> I( {\mathscr {N}}_{A\rightarrow B_1} ), \\ &  r_2 > I( {\mathscr {N}}_{A\rightarrow B_2} ). \\ \end{array}\right. } \end{aligned}$$

Here, the quantum mutual information of channel is defined in (2.13).

### Theorem 6

(Capacity region for 2-party Quantum Broadcast Channel Simulation). Let ${\mathscr {N}}_{A\rightarrow B_1 B_2}$ be a quantum broadcast channel. The capacity region of simulating ${\mathscr {N}}_{A\rightarrow B_1 B_2}$ is given by

5.8 $$\begin{aligned} {\mathcal {C}}({\mathscr {N}}_{A\rightarrow B_1 B_2})&= \left\{ \vec {r} \in \mathbb {R}^2: r_1 + r_2 \ge I({\mathscr {N}}_{A\rightarrow B_1 B_2} ), \, r_1 \ge I( {\mathscr {N}}_{A\rightarrow B_1}), \, r_2 \ge I( {\mathscr {N}}_{A\rightarrow B_2} ) \right\} . \end{aligned}$$

### Remark 8

Though our target is to simulate the broadcast channel ${\mathscr {N}}_{A\rightarrow B_1 B_2}$, the established achievability given in Theorems 5 and 6 are actually stronger for the *coherent feedback simulation* of ${\mathscr {N}}_{A\rightarrow B_1 B_2}$; namely, we are able to simulate the isometry ${\mathscr {V}}_{A\rightarrow B_1 B_2 E}$, where the complementary part *E* is retained at Sender [3, 119].

### Remark 9

The minimax identity given in Proposition 2-(iii) shows that the error-exponent function of channel in Theorem 5 can be viewed as the error-exponent function for the channel output state induced by the worst-case input state. Namely, the error terms (i.e. $\delta _{1,2}$, $\delta _1$, and $\delta _2$ in Theorem 5) for channel simulation are dominated by the worst-case input states, respectively.

### Proof of Theorem 6

The achievability directly follows from the exponential decreases of error given in Theorem 5, and noting that $\lim _{n\in \infty } \frac{1}{n} \log k_n = 0$.

For the converse, we first note that the requirements on the separate rates $r_1$ and $r_2$ follow from the converse of the respective single-sender single-receiver reverse Shannon theorem [3, 3, 3]. That is, for an asymptotically vanishing error we need5.9For the rate sum constraint $r_1+r_2$ the argument is similar as in the classical case [120, Theorem 36] and a simple version for the first order asymptotics as needed here is as follows. The idea is to analyze the correlations between the purifying reference system *R* and the respective receivers in terms of the multipartite mutual information. Any quantum broadcast simulation protocol applies an encoder on the sender’s side, uses classical communication at a rate $r_i$ from the sender to the *i*-th receiver’s side, and then applies local decoders on the receiver’s end. The multipartite mutual information is monotone under local operations at the receiver’s end (similarly as the mutual information) and increases at most by $nr_i$ by sending bits at a rate $r_i$ to the *i*-the receiver, as shown by the following dimension bound (cf. the textbook methods [120])5.10for any classical-quantum state $\sigma _{X_1X_2C_1C_2}$, classical on systems $X_1X_2$. However, at the end of any $\varepsilon$-good protocol (Definition 2) for a purified i.i.d. input state $(\psi ^\rho _{AR})^{\otimes n}$, the output state on the relevant systems has to be $\varepsilon$-close in variational distance to $\left[ ({\mathscr {N}}_{A\rightarrow B_1B_2}\otimes {\mathcal {I}}_R)(\psi ^\rho _{AR})\right] ^{\otimes n}$. As the continuity of the multipartite mutual information is inherited from the continuity of the von Neumann entropy (see, e.g., [121] for tight estimates), we need at the end of the protocol that the multipartite mutual information on the relevant systems obeys5.11where we also employed that the multipartite mutual information is additive on tensor product states. Since this has to hold for any i.i.d. input states $\rho _A^{\otimes n}$, the claim follows from taking the limit $\varepsilon \rightarrow 0$ for asymptotically perfect protocols. $\square$

### Proposition 3

(Error exponent). Let ${\mathscr {N}}_{A\rightarrow B_1 B_2}$ be a quantum broadcast channel. There exists a sequence of $(n \vec {r}, \varepsilon _n)$ Quantum Broadcast Channel Simulation protocol for ${\mathscr {N}}_{A\rightarrow B_1 B_2}^{\otimes n}$ satisfying achievable error exponent bound:5.12Moreover, the overall error exponent in the lower bound is positive if and only if $\vec {r}$ is in the interior of the capacity region ${\mathcal {C}}({\mathscr {N}})$ given in Eq. (5.8).

### Remark 10

The optimal error exponent (under *channel purified distance*) was established for point-to-point quantum channel simulation [45]. Even for the special case of point-to-point quantum channel simulation, it is not clear whether the error exponent given in Proposition 3 or the result in [45] will give a better error exponent under diamond norm due to the fundamental different expressions of the error-exponent functions.

Our intention in this paper, however, is not to derive the optimal error exponent, but to devise a machinery for analyzing one-shot quantum broadcast channel simulation and the corresponding capacity region.

The overall error exponent for Quantum Broadcast Channel Simulation is positive if and only if the classical communication cost $\vec {r}$ is in the interior of the capacity region ${\mathcal {C}}({\mathscr {N}})$ as shown in Theorem 5 and Proposition 3. One may wonder if the broadcast channel simulation is still achievable as $\vec {r}$ asymptotically converges to the boundary ${\mathcal {C}}({\mathscr {N}})$? Note that in this scenario the overall error exponent will vanish asymptotically. In the following Proposition 4, we show that the broadcast channel simulation is still achievable once $\vec {r}$ converges to the boundary ${\mathcal {C}}({\mathscr {N}})$ of speed $\Theta (n^{-t})$ for some $t\in (0,1/2)$. We call such $\Theta (n^{-t})$ a *strictly moderate sequence* [23, 44, 122, 123] and such a study as a *moderate deviation analysis* (compared to the *large deviation analysis* given in Theorem 5 and Proposition 3, in which $\vec {r}$ is bounded away from the boundary of ${\mathcal {C}}({\mathscr {N}})$).

### Proposition 4

(Achievability for moderate deviation analysis). Let ${\mathscr {N}}_{A\rightarrow B_1 B_2}$ be a quantum broadcast channel on finite-dimensional Hilbert spaces.

For any sequence $\vec {r}_n \,{:=}\, (r_{1,n}, r_{2,n})\in {\mathcal {C}}({\mathscr {N}}_{A\rightarrow B_1 B_2})$ satisfying

5.13 $$\begin{aligned} a_n \,{:=}\, \min \left\{ \frac{r_{1,n} - I({\mathscr {N}}_{A\rightarrow B_1})}{\sqrt{V({\mathscr {N}}_{A\rightarrow B_1})}}, \frac{r_{2,n} - I({\mathscr {N}}_{A\rightarrow B_2})}{\sqrt{V({\mathscr {N}}_{A\rightarrow B_1})}} \frac{r_{1,n} + r_{2,n} - I({\mathscr {N}}_{A\rightarrow B_1 B_2})}{\sqrt{V({\mathscr {N}}_{A\rightarrow B_1 B_2})}} \right\} = \Theta (n^{-t}) \end{aligned}$$

for some $t\in (0,1/2)$, then there exists a sequence of $(n \vec {r}_n, \varepsilon _n)$ Quantum Broadcast Channel Simulation protocols for ${\mathscr {N}}_{A\rightarrow B_1 B_2}^{\otimes n}$ such that

5.14 $$\begin{aligned} \liminf _{n\rightarrow \infty } -\frac{1}{n a_n^2} \log \varepsilon _n \ge \frac{1}{4}. \end{aligned}$$

### Remark 11

For the special case of point-to-point quantum channel simulation, Proposition 4 translates to the following scenario: given $\varepsilon _n = 2^{- n a_n^2}$, then there exists a sequence of $(nr_n, \varepsilon _n)$-quantum channel simulation protocols for ${\mathscr {N}}_{A\rightarrow B}^{\otimes n}$ such that $r_n \le I({\mathscr {N}}) + 2\sqrt{V({\mathscr {N}}) }\cdot a_n$. Such an achievable rate for classical communication cost coincides with the result given in [44] (under *channel purified distance*). We expect that the best achievable moderate deviation expansion of the classical communication cost under diamond norm is $I({\mathscr {N}}) + \sqrt{2V({\mathscr {N}})}\cdot a_n$, which is left as a future work.

The proof of Proposition 4 follows from the achievability given in Theorem 5, the properties of error-exponent function (Proposition 2), and the standard moderate deviation analysis given in [23, 85, 111].

### Proof

Let us first consider the unipartite case, i.e. ${\mathscr {N}}_{A\rightarrow B}$ provided that $V({\mathscr {N}}_{A\rightarrow B}) >0$. Let

5.15 $$\begin{aligned} r_n&\,{:=}\, I({\mathscr {N}}) + a_n, \end{aligned}$$

where $(a_n)_{n\in \mathbb {N}}$ is any positive sequence satisfying $a_n = \Theta (n^{-t})$ for some $t\in (0,1/2)$. We recall Proposition 2-(iv):

5.16 $$\begin{aligned} \liminf _{n\rightarrow \infty }\frac{E_{r_n}({\mathscr {N}})}{a_n^2}&\ge \frac{1}{2V({\mathscr {N}})}. \end{aligned}$$

Then by Theorem 5 (with $L=1$), we estimate the error probability for simulating ${\mathscr {N}}_{A\rightarrow B}^{\otimes n}$ using rate $n r_n$ as

5.17 $$\begin{aligned} \liminf _{n\rightarrow \infty } -\frac{1}{n a_n^2 }\log \varepsilon ({\mathscr {N}}_{A\rightarrow B}^{\otimes n})&\ge \liminf _{n\rightarrow \infty } -\frac{1}{n a_n^2 }\log \left\{ k_n \sqrt{ 2^{-E_{n r_n}({\mathscr {N}}^{\otimes n}) } } \right\} \end{aligned}$$

5.18 $$\begin{aligned}&= \liminf _{n\rightarrow \infty }\left\{ \frac{E_{n r_n}({\mathscr {N}}^{\otimes n}) }{2 n a_n^2} - \log \frac{k_n}{n a_n^2} \right\} \end{aligned}$$

5.19 $$\begin{aligned}&= \liminf _{n\rightarrow \infty }\left\{ \frac{E_{r_n}({\mathscr {N}}) }{2 a_n^2} - \log \frac{k_n}{n^{1 - 2t}} \right\} \end{aligned}$$

5.20 $$\begin{aligned}&\ge \frac{1}{4 V({\mathscr {N}}_{A\rightarrow B})}, \end{aligned}$$

where the last line holds for any polynomial pre-factor $k_n$ for any $t\in (0,1/2)$. This leads to our assertion of the moderate deviation achievability for simulating point-to-point channel ${\mathscr {N}}_{A\rightarrow B}$ via re-scaling $r_n = I({\mathscr {N}}) + a_n$ by $r_n = I({\mathscr {N}}) + \sqrt{V({\mathscr {N}})} \cdot a_n$.

Similar reasoning straightforwardly applies to the multipartite case, i.e. for any non-empty subset $S\subseteq [L]$ (with fixed $L \in \mathbb {N}$), we have

5.21 $$\begin{aligned} \liminf _{n\rightarrow \infty }\frac{E_{r_n}({\mathscr {N}}_{A\rightarrow B_S})}{a_n^2}&\ge \frac{1}{2V({\mathscr {N}}_{A\rightarrow B_S})}, \end{aligned}$$

and thus

5.22 $$\begin{aligned} \liminf _{n\rightarrow \infty } -\frac{1}{n a_n^2 }\log \varepsilon ({\mathscr {N}}_{A\rightarrow B_S}^{\otimes n})&\ge \frac{1}{4 V({\mathscr {N}}_{A\rightarrow B_S})}. \end{aligned}$$

Combined with Theorem 5, the overall asymptotic error decay will be dominated by the worst channel output subset *S*. This concludes the proof. $\square$

We now present the proof of bipartite quantum broadcast channel simulation.

### Proof of Theorem 5

Before diving into the proof, let us first elaborate on the proof structure. Our main technique relies on the multipartite Quantum State Splitting established in Sect. 4 and the *Post-Selection Technique* [69]. Note that the idea of unipartite Quantum State Splitting and the Post-Selection Technique have been used to prove point-to-point quantum channel simulation [3]. Unlike [3] working with the smooth entropy formalism, we will demonstrate how the Post-Selection Technique can work with Rényi information measures by employing certain properties shown in Lemmas 1 and 2.

We start with the *de Finetti* type input state: $\zeta _{AR}^n = \int \psi _{AR}^{\otimes n} \, \textrm{d}(\psi _{AR})$, where $\psi _{AR} \in {\mathcal {S}}({\textsf{A}}\otimes {\textsf{R}})$ is a pure state, and the integration is with respect to the Haar measure on the unitary group acting on ${\textsf{A}}\otimes {\textsf{R}}$. Moreover, we denote by $\zeta _{ARR'}^n$ a purification of $\zeta _{AR}^n$. Let ${\mathscr {U}}_{A\rightarrow B_1 B_2 E}$ be a Stinespring dilation of the broadcast channel ${\mathscr {N}}_{A\rightarrow B_1 B_2}$. Then, Sender first simulate a local isometry ${\mathscr {N}}_{A\rightarrow B_1 B_2}$ at her side to obtain the state

5.23 $$\begin{aligned} \zeta _{B_1 B_2 E R R'}^n \,{:=}\, \left( {\mathscr {U}}_{A\rightarrow B_1 B_2 E}^{\otimes n} \otimes \textrm{id}_{RR'} \right) \zeta _{ARR'}^n. \end{aligned}$$

Next, we apply the 2-party Quantum State Splitting protocol (Theorem 3) with registers $A\leftarrow E$, $A_1'\leftarrow B_1$, $A_2'\leftarrow B_2$, and $R\leftarrow RR'$, to send the $B_1$-part to Receiver 1 via $n r_1$ bits of classical communication and send the $B_2$-part to Receiver 2 via $n r_2$ bits of classical communication. The pre-shared entanglement used in the protocol is many copies of $|\zeta ^n\rangle _{B_1 \bar{B}_1}$ and $|\zeta ^n\rangle _{B_2 \bar{B}_2}$, where the first Receiver holds register $\bar{B}_1^n$ and the second Receiver holds register $\bar{B}_2^n$. The above procedure is embodied by a simulated isometry $\widehat{{\mathscr {U}}}_{A\rightarrow B_1 B_2 E}^n$ acting on the state $\zeta _{ARR'}^n$. Then, the resulting error, denoted by $\varepsilon '(\zeta ^n)$, is

5.24 $$\begin{aligned} \frac{1}{2} \left\| \left( \widehat{{\mathscr {U}}}_{A\rightarrow B_1 B_2 E}^n - {\mathscr {U}}_{A\rightarrow B_1 B_2 E}^{\otimes n} \right) (\zeta _{ARR'}^n)\right\| _1&\le \varepsilon '(\zeta ^n), \end{aligned}$$

5.25 $$\begin{aligned} \varepsilon '(\zeta ^n)&\,{:=}\, \sqrt{ 2 \cdot \delta _{1,2}'(\zeta ^n) + \delta _1'(\zeta ^n) + \delta _2'(\zeta ^n) }, \end{aligned}$$

5.26 $$\begin{aligned} \delta _{1,2}'(\zeta ^n)&\,{:=}\, 2^{- E_{nr_1+nr_2}(B_1: B_2 : R R')_{({\mathscr {N}}^{\otimes n} \otimes \textrm{id})(\zeta ^n)} }, \end{aligned}$$

5.27 $$\begin{aligned} \delta _1'(\zeta ^n)&\,{:=}\, 2^{- E_{nr_1}(B_1: RR')_{({\mathscr {N}}^{\otimes n} \otimes \textrm{id})(\zeta ^n)} }, \end{aligned}$$

5.28 $$\begin{aligned} \delta _2'(\zeta ^n)&\,{:=}\, 2^{- E_{nr_2}(B_2: RR')_{({\mathscr {N}}^{\otimes n} \otimes \textrm{id})(\zeta ^n)} }, \end{aligned}$$

where the error-exponent function is defined in Eq. (2.24). Moreover, by tracing out the system $E^n$ and the monotonicity of trace distance [124], we obtain the bound:

5.29 $$\begin{aligned} \frac{1}{2} \left\| \left( \widehat{{\mathscr {N}}}_{A\rightarrow B_1 B_2}^{n} - {\mathscr {N}}_{A\rightarrow B_1 B_2}^{\otimes n} \right) (\zeta _{ARR'}^n)\right\| _1 \le \varepsilon '(\zeta ^n). \end{aligned}$$

Here, the channel $\widehat{{\mathscr {N}}}_{A\rightarrow B_1 B_2}^{n}$ is the effectively simulated proximity to our target ${\mathscr {N}}_{A\rightarrow B_1 B_2}^{\otimes n}$.

By using the Post-Selection Technique (Proposition 2), this guarantees that

5.30 $$\begin{aligned} \begin{aligned} \frac{1}{2} \left\| \widehat{{\mathscr {N}}}_{A\rightarrow B_1 B_2}^{n} - {\mathscr {N}}_{A\rightarrow B_1 B_2}^{\otimes n} \right\| _\diamond \le (n+1)^{|{\textsf{A}}|^2 - 1} \varepsilon '(\zeta ^n). \end{aligned} \end{aligned}$$

It remains to remove the auxiliary system $R'$ and to upper bound the error $\varepsilon '(\zeta ^n)$ with the one dominated by the worst-case state $\psi _{AR}^{\otimes n}$ in the mixture of the de Finetti type state $\zeta _{AR}^n$.

Note that we can assume $|{\textsf{R}}'|\le (n+1)^{|{\textsf{A}}|^2-1}$ by Fact 2. Further, Lemma 1 shows that, for each $\alpha >0$,

5.31 $$\begin{aligned}&I_\alpha (B_1 : B_2 : RR')_{({\mathscr {N}}^{\otimes n} \otimes \textrm{id})(\zeta ^n)}\nonumber \\&\le I_\alpha (B_1 : B_2 : R)_{({\mathscr {N}}^{\otimes n} \otimes \textrm{id})(\zeta ^n)} + \frac{2\alpha }{\alpha -1} \log \left[ (n+1)^{|{\textsf{A}}|^2 - 1} \right] , \end{aligned}$$

which by the definition of the error-exponent function in Eq. (2.24), in turn, implies that

5.32 $$\begin{aligned}&E_{nr_1 + nr_2}(B_1: B_2 : RR')_{({\mathscr {N}}^{\otimes n} \otimes \textrm{id})(\zeta ^n)} \nonumber \\&\ge E_{nr_1 + nr_2}(B_1: B_2 : R)_{({\mathscr {N}}^{\otimes n} \otimes \textrm{id})(\zeta ^n)} - 2 \log \left[ (n+1)^{|{\textsf{A}}|^2 - 1} \right] ; \end{aligned}$$

likewise for $E_{nr_1 }(B_1: RR')_{({\mathscr {N}}^{\otimes n} \otimes \textrm{id})(\zeta ^n)}$ and $E_{nr_2}( B_2: RR')_{({\mathscr {N}}^{\otimes n} \otimes \textrm{id})(\zeta ^n)}$.

Using Carathéodory’s type theorem (Fact 3), we can write

5.33 $$\begin{aligned} \zeta _{AR}^n = \sum \nolimits _{i\in {\mathcal {I}}} p_i (\psi _{AR}^i)^{\otimes n} \end{aligned}$$

for some pure state $\psi _{AR}^i$, finite set ${\mathcal {I}}$ with $|{\mathcal {I}}| = (n+1)^{2|{\textsf{A}}||{\textsf{R}}|-2}$, and probability distribution $\{p_i\}_{i\in {\mathcal {I}}}$. Then, Lemma 2 with $L=2$ and the additivity of Rényi information (Proposition 1-(b)) show that

5.34 $$\begin{aligned} I_\alpha (B_1 : B_2 : R)_{({\mathscr {N}}^{\otimes n} \otimes \textrm{id})(\sum _i p_i (\psi ^i)^{\otimes n})}&\le \sum \nolimits _{i\in {\mathcal {I}}} p_i I_\alpha (B_1 : B_2 : R)_{({\mathscr {N}}^{\otimes n} \otimes \textrm{id})((\psi ^i)^{\otimes n})}\nonumber \\&+ 2 \log \left[ (n+1)^{2|{\textsf{A}}||{\textsf{R}}| - 2} \right] \end{aligned}$$

5.35 $$\begin{aligned}&= \sum \nolimits _{i\in {\mathcal {I}}} p_i n I_\alpha (B_1 : B_2 : R)_{({\mathscr {N}} \otimes \textrm{id})(\psi ^i)}\nonumber \\&+ 2 \log \left[ (n+1)^{|{\textsf{A}}||{\textsf{R}}| - 2} \right] \end{aligned}$$

5.36 $$\begin{aligned}&\le \max _{i\in {\mathcal {I}}} n I_\alpha (B_1 : B_2 : R)_{({\mathscr {N}}\otimes \textrm{id})(\psi ^i)}\nonumber \\ 7 + 2 \log \left[ (n+1)^{2|{\textsf{A}}||{\textsf{R}}| - 2} \right] \end{aligned}$$

5.37 $$\begin{aligned}&\le n I_\alpha ({\mathscr {N}}_{A\rightarrow B_1 B_2})+ 2 \log \left[ (n+1)^{2|{\textsf{A}}||{\textsf{R}}| - 2} \right] . \end{aligned}$$

This then implies that

5.38 $$\begin{aligned} E_{nr_1 + nr_2}(B_1: B_2 : R)_{({\mathscr {N}}^{\otimes n} \otimes \textrm{id})(\zeta ^n)} \ge n E_{r_1 + r_2}({\mathscr {N}}_{A\rightarrow B_1 B_2}) - \log \left[ (n+1)^{2|{\textsf{A}}||{\textsf{R}}| - 2} \right] . \end{aligned}$$

Similar bounds hold for $E_{nr_1 }(B_1: R)_{({\mathscr {N}}^{\otimes n} \otimes \textrm{id})(\zeta ^n)}$ and $E_{nr_2}( B_2: R)_{({\mathscr {N}}^{\otimes n} \otimes \textrm{id})(\zeta ^n)}$ as well.

Lastly, combining the prefactors incurred in Eqs. (5.30), (5.32), and (5.38), we conclude the proof from Eq. (5.25) with the overall polynomial prefactor:

5.39 $$\begin{aligned} (n+1)^{\frac{|{\textsf{A}}|^2-1}{2}} (n+1)^{|{\textsf{A}}|^2-1}(n+1)^{{|{\textsf{A}}||{\textsf{R}}|-1}} \le (n+1)^{\frac{5}{2} ( |{\textsf{A}}|^2-1 ) } =: k_n. \end{aligned}$$

$$\square $$

The scenario of simulation arbitrary *L*-party quantum broadcast channel follows the same proof as in the bipartite case (Theorem 5) and the multipartite Quantum State Splitting established in Theorem 4.

### Theorem 7

(Non-asymptotic achievability for *L*-party Quantum Broadcast Channel Simulation). Let ${\mathscr {N}}_{A\rightarrow B_1 B_2\ldots B_L}$ be an *L*-receiver quantum broadcast channel. For any integer *n*, there exists an $(n \vec {r}, \varepsilon )$ Quantum Broadcast Channel Simulation protocol for ${\mathscr {N}}_{A\rightarrow B_1 B_2\ldots B_L}^{\otimes n}$ satisfying

5.40 $$\begin{aligned} \varepsilon&\le k_n \cdot \sqrt{ \sum \nolimits _{\varnothing \ne S \subseteq [L]} 2^{|S|} \cdot 2^{- n E_{r_S}({\mathscr {N}}_{A\rightarrow B_S}) } }, \end{aligned}$$

where the error-exponent functions $E_{r_S}({\mathscr {N}}_{A\rightarrow B_S})$ are defined in Eq. (2.25), the polynomial prefactor is $k_n \,{:=}\, (n+1)^{\frac{3+L}{2} (|{\textsf{A}}|^2-1) }$, $r_S\,{:=}\, \sum _{ \ell \in S } r_\ell$, and $B_S$ denotes systems $B_\ell$ for all $\ell \in S$.

### Theorem 8

(Capacity region for *L*-party Quantum Broadcast Channels Simulation). Let ${\mathscr {N}}_{A\rightarrow B_1 B_2\ldots B_L}$ be an *L*-receiver quantum broadcast channel. The capacity region of simulating ${\mathscr {N}}_{A\rightarrow B_1 B_2\ldots B_L}$ is given by

5.41 $$\begin{aligned} {\mathcal {C}}({\mathscr {N}}_{A\rightarrow B_1 \ldots B_L}) = \left\{ \vec {r} \in \mathbb {R}^L: \sum \nolimits _{ \ell \in S } r_\ell \ge I({\mathscr {N}}_{A\rightarrow B_S}), \forall S \subseteq [L] \right\} , \end{aligned}$$

where $I({\mathscr {N}}_{A\rightarrow B_S})$ is defined in (2.13), and $B_S$ denotes systems $B_\ell$ for all $\ell \in S$.

Moreover, the lower bound to the error exponent and the achievability of moderate deviations as stated in Propositions 3 and 4 also immediately hold for *L*-receiver broadcast channel simulation.

## Appendix A. Technical Lemmas

### Fact 1

(Uhlmann’s theorem [125, 126, 41, Lemma 2.2]) Let $\psi _{AB} = |\psi \rangle \langle \psi |_{AB}$ and $\varphi _{AC} = |\varphi \rangle \langle \varphi |_{AC}$ be two pure quantum states. Then, there exists an isometry ${\mathscr {V}}_{B\rightarrow C}$ satisfying

A.1 $$\begin{aligned}&\frac{1}{2}\Vert \psi _{A} - \varphi _{A} \Vert _1 \le \varepsilon \Rightarrow \frac{1}{2}\Vert {\mathscr {V}}_{B\rightarrow C}( \psi _{AB} ) - \varphi _{AC} \Vert _1\nonumber \\&= \textrm{P}({\mathscr {V}}_{B\rightarrow C}( \psi _{AB} ), \varphi _{AC} ) \le \sqrt{ 2\varepsilon - \varepsilon ^2 } \le \sqrt{ 2\varepsilon }. \end{aligned}$$

### Lemma 2.3

(Dimension bound) For any states $\rho _{ABC} \in {\mathcal {S}}({\textsf{A}} \otimes {\textsf{B}} \otimes {\textsf{C}})$, $\tau _{A} \in {\mathcal {S}}({\textsf{A}})$, and $\alpha >0$, we have

A.2 $$\begin{aligned} I_\alpha \left( \rho _{ABC} \,\Vert \, \tau _A \right) \le I_\alpha \left( \rho _{AB} \,\Vert \, \tau _A \right) + \frac{2\alpha }{\alpha -1} \log |{\textsf{C}}|. \end{aligned}$$

### Proof

For every $\varepsilon >0$, let $\sigma _B \in {\mathcal {S}}({\textsf{B}})$ be a density operator satisfying

A.3 $$\begin{aligned} D_\alpha (\rho _{AB} \,\Vert \, \tau _A \otimes \sigma _B) \le I_\alpha \left( \rho _{AB} \,\Vert \, \tau _A \right) + \varepsilon , \end{aligned}$$

and denote by ${\tilde{\sigma }}_{BC} \,{:=}\, \sigma _B \otimes \frac{\mathbb {1}_C}{|{\textsf{C}}|}$ for short. Note that $\rho _{ABC} \le |{\textsf{C}}| \cdot \rho _{AB} \otimes \mathbb {1}_C$, which implies

A.4 $$\begin{aligned} \frac{1}{|{\textsf{C}}|^2} \rho _{ABC} \le \rho _{AB} \otimes \frac{\mathbb {1}_C}{|{\textsf{C}}|}. \end{aligned}$$

Then,

A.5 $$\begin{aligned} \frac{1}{|{\textsf{C}}|^2} \left( \tau _A \otimes {\tilde{\sigma }}_{BC} \right) ^{\frac{1-\alpha }{2\alpha }} \rho _{ABC} \left( \tau _A \otimes {\tilde{\sigma }}_{BC} \right) ^{\frac{1-\alpha }{2\alpha }} \le \left( \tau _A \otimes {\tilde{\sigma }}_{BC} \right) ^{\frac{1-\alpha }{2\alpha }} \rho _{AB} \otimes \frac{\mathbb {1}_C}{|{\textsf{C}}|} \left( \tau _A \otimes {\tilde{\sigma }}_{BC} \right) ^{\frac{1-\alpha }{2\alpha }}. \end{aligned}$$

This implies, for all $\alpha > 0$,

A.6 $$\begin{aligned}&\frac{1}{|{\textsf{C}}|^{2}} \left\| \left( \tau _A \otimes {\tilde{\sigma }}_{BC} \right) ^{\frac{1-\alpha }{2\alpha }} \rho _{ABC} \left( \tau _A \otimes {\tilde{\sigma }}_{BC} \right) ^{\frac{1-\alpha }{2\alpha }} \right\| _\alpha \nonumber \\&\le \left\| \left( \tau _A \otimes {\tilde{\sigma }}_{BC} \right) ^{\frac{1-\alpha }{2\alpha }} \rho _{AB} \otimes \frac{\mathbb {1}_C}{|{\textsf{C}}|} \left( \tau _A \otimes {\tilde{\sigma }}_{BC} \right) ^{\frac{1-\alpha }{2\alpha }} \right\| _\alpha . \end{aligned}$$

By invoking the definition of the sandwiched Rényi divergence $D_\alpha (\rho \,\Vert \,\sigma ) \,{:=}\, \frac{\alpha }{\alpha -1} \log \left\| \sigma ^{\frac{1-\alpha }{2\alpha }} \rho \sigma ^{\frac{1-\alpha }{2\alpha }} \right\| _\alpha$, we have

A.7 $$\begin{aligned} D_\alpha \left( \rho _{ABC} \,\Vert \, \tau _A \otimes {\tilde{\sigma }}_{BC} \right) - \frac{2\alpha }{\alpha -1} \log |{\textsf{C}}|&\le D_\alpha \left( \left. \rho _{AB} \otimes \frac{\mathbb {1}_C}{|{\textsf{C}}|} \,\right\| \, \tau _A \otimes {\tilde{\sigma }}_{BC} \right) \end{aligned}$$

A.8 $$\begin{aligned}&= D_\alpha \left( \rho _{AB} \,\Vert \, \tau _A \otimes {\sigma }_{B} \right) \end{aligned}$$

A.9 $$\begin{aligned}&\le I_\alpha \left( \rho _{AB} \,\Vert \, \tau _A \right) +\varepsilon , \end{aligned}$$

where we have used the fact that the sandwiched Rényi divergence remains identical by appending a state.

Lastly, by definition, noting that

A.10 $$\begin{aligned} D_\alpha \left( \rho _{ABC} \,\Vert \, \tau _A \otimes {\tilde{\sigma }}_{BC} \right) \ge I_\alpha \left( \rho _{ABC} \,\Vert \, \tau _A \right) , \end{aligned}$$

and letting $\varepsilon \rightarrow 0$ conclude the proof. $\square$

### Lemma 2.4

(Convexity) Let *L* be any integer and ${\mathcal {I}}$ be any finite set. Let $\rho _{A_1A_2\ldots A_L E} \,{:=}\, \sum _{i\in {\mathcal {I}}} p_i \rho _{A_1A_2\ldots A_L E}^{i}$ and $\tau _{A_\ell } = \sum _{i\in {\mathcal {I}}} p_i \tau _{A_{\ell }}^{i}$, $\ell \in [L]$ be statistical mixtures of states for any $p_i>0$, $\sum _{i\in {\mathcal {I}}} p_i = 1$. Then, the following holds for every $\alpha \ge 1/2$,

A.11 $$\begin{aligned} \begin{aligned} I_\alpha ( \rho _{A_1\ldots A_L E} \,\Vert \otimes _{\ell \in [L]} \tau _{A_\ell } )&\le \sum \nolimits _{i\in {\mathcal {I}} } p_i I_\alpha \left( \rho _{A_1\ldots A_L E}^{i} \,\Vert \otimes _{\ell \in [L]} \tau _{A_\ell }^{i}\right) + L \cdot H(\{p_i\}_{i\in {\mathcal {I}}}) \\  &\le \sum \nolimits _{i \in {\mathcal {I}} }p_i I_\alpha \left( \rho _{A_1\ldots A_L E}^{i} \,\Vert \otimes _{\ell \in [L]} \tau _{A_\ell }^{i}\right) + L \log |{\mathcal {I}}|. \end{aligned} \end{aligned}$$

Here, $H(\{p_i\}_{i\in {\mathcal {I}}}) \,{:=}\, - \sum _{i\in {\mathcal {I}}} p_i \log p_i$ denotes the Shannon entropy.

### Proof

Below, the subscript $\ell$ runs over all [*L*] and the subscript *i* runs over all ${\mathcal {I}}$ if we do not specify it. For each $i\in {\mathcal {I}}$ and every $\varepsilon >0$, we let $\sigma _E^{i} \in {\mathcal {S}}( {\textsf{E}})$ to be a state satisfying

A.12 $$\begin{aligned} D_\alpha \left( \rho _{A_1\ldots A_L E}^{i} \,\Vert \otimes _{\ell } \tau _{A_{\ell }}^{i} \otimes \sigma _E^{i}\right) \le I_\alpha \left( \rho _{A_1\ldots A_L E}^i \,\Vert \, \otimes _{\ell } \tau _{A_\ell }^{i} \right) + \varepsilon . \end{aligned}$$

Then, by the definition of the sandwiched Rényi divergence, we have

A.13 $$\begin{aligned}&\sum \nolimits _i p_i D_\alpha \left( \rho _{A_1\ldots A_L E}^{i} \,\Vert \otimes _{\ell } \tau _{A_{\ell }}^{i} \otimes \sigma _E^{i}\right) - L \cdot p_i \log p_i \end{aligned}$$

A.14 $$\begin{aligned}&= \sum \nolimits _i p_i D_\alpha \left( \rho _{A_1\ldots A_L E}^{i} \,\Vert \, p_i \tau _{A_{1}}^i \otimes \cdots \otimes p_i \tau _{A_{L}}^i \otimes \sigma _E^{i}\right) \end{aligned}$$

A.15 $$\begin{aligned}&\overset{\text {(a)}}{\ge } D_\alpha \left( \sum \nolimits _i p_i\rho _{A_1\ldots A_L E}^{i} \,\Vert \sum \nolimits _i p_i \tau _{A_{1}}^i \otimes \cdots \otimes p_i \tau _{A_{L}}^i \otimes p_i \sigma _E^{i}\right) \end{aligned}$$

A.16 $$\begin{aligned}&\overset{\text {(b)}}{\ge } D_\alpha \left( \sum \nolimits _i p_i\rho _{A_1\ldots A_L E}^{i} \,\Vert \sum \nolimits _{i_1} p_{i_1} \tau _{A_{1}}^{i_1} \otimes \cdots \otimes \sum \nolimits _{i_L} p_{i_L} \tau _{A_{L}}^{i_1} \otimes \sum \nolimits _i p_i \sigma _E^{i}\right) \end{aligned}$$

A.17 $$\begin{aligned}&= D_\alpha \left( \rho _{A_1\ldots A_L E} \,\Vert \tau _{A_{1}} \otimes \cdots \otimes \tau _{A_{L}} \otimes \sum \nolimits _i p_i \sigma _E^{i}\right) \end{aligned}$$

A.18 $$\begin{aligned}&\ge I_\alpha ( \rho _{A_1\ldots A_L E} \,\Vert \otimes _{\ell \in [L]} \tau _{A_\ell } ), \end{aligned}$$

where (a) follows from the convexity of the sandwiched Rényi divergence [76, 77, 127–129, Theorem 3.16], and (b) follows from the monotonically non-increasing map $\sigma \mapsto D_\alpha (\rho \,\Vert \,\sigma )$ [76, Proposition 4], [129, Lemma 3.24], and for each $\ell \in [L]$, $p_i \tau _{A_\ell }^i \le \sum \nolimits _{ i_\ell \in [L]} p_{i_\ell } \tau _{A_\ell }^{{i}_\ell }$. By letting $\varepsilon \rightarrow 0$, we conclude the proof. $\square$

### Proposition 2.2

(Properties of error-exponent function) For any multipartite state $\rho _{A_1 A_2 \ldots A_L B}$ and any quantum broadcast channel ${\mathscr {N}}_{A\rightarrow B_1 B_2 \ldots B_L}$, the error-exponent function satisfies the following properties.

(i) (Positivity) For any $r>0$,
  A.19 $$\begin{aligned} E_r (\rho _{A_1 A_2 \ldots A_L B} \, \Vert \otimes _{\ell \in [L]} \tau _{A_\ell })> 0&\Longleftrightarrow r > I(\rho _{A_1 A_2 \ldots A_L B} \, \Vert \otimes _{\ell \in [L]} \tau _{A_\ell }); \end{aligned}$$
  A.20 $$\begin{aligned} E_r ({\mathscr {N}}_{A\rightarrow B_1 B_2 \ldots B_L})> 0&\Longleftrightarrow r > I( {\mathscr {N}}_{A\rightarrow B_1 B_2 \ldots B_L} ). \end{aligned}$$
(ii) (Additivity) For any integer $n \in \mathbb {N}$ and $r>0$,
  A.21 $$\begin{aligned} E_{nr} \left( \rho _{A_1 A_2 \ldots A_L B}^{\otimes n} \, \Vert \otimes _{\ell \in [L]} \tau _{A_\ell }^{\otimes n} \right) = n E_{{r}}\left( \rho _{A_1 A_2 \ldots A_L B} \, \Vert \otimes _{\ell \in [L]} \tau _{A_\ell } \right) . \end{aligned}$$
(iii) (A minimax identity and saddle-point) Provided that the underlying Hilbert spaces are all finite dimensional, for any $r>0$, there exist a saddle-point $(\psi ,\alpha ) \in {\mathcal {S}}({\textsf{A}}\otimes {\textsf{R}}) \times [1,2]$ [114, §36] such that
  A.22 $$\begin{aligned} E_r({\mathscr {N}}_{A\rightarrow B_1 B_2 \ldots B_L})&= \inf _{\psi _{AR}} E_r(B_1: B_2: \cdots B_L: R)_{({\mathscr {N}}\otimes \textrm{id}_R)(\psi )} \end{aligned}$$
  A.23 $$\begin{aligned}&= \frac{\alpha -1}{\alpha } \left( r - I_\alpha (B_1:B_2\cdots B_L: R)_{({\mathscr {N}}\otimes \textrm{id}_R)(\psi )} \right) . \end{aligned}$$
(iv) (Limiting behavior) Provided that the underlying Hilbert spaces are all finite dimensional, then for any sequence $r_n \,{:=}\, I\left( {\mathscr {N}}_{A\rightarrow B_1 B_2\ldots B_L}\right) + a_n$ satisfying $a_n \downarrow 0$, we have
  A.24 $$\begin{aligned} \liminf _{n\rightarrow \infty }\frac{E_{r_n}({\mathscr {N}})}{a_n^2}&\ge \frac{1}{2V({\mathscr {N}}_{A\rightarrow B_1 B_2\ldots B_L})}. \end{aligned}$$

### Proof

Items (i), and (ii) directly follows from Proposition 1-(a) and (b).

By Proposition (c) and (d), the objective function

A.25 $$\begin{aligned} (\rho _A, \alpha ) \mapsto \frac{\alpha -1}{\alpha } \left( r - I_{\alpha }(B:R)_{{\mathscr {N}}(\psi _{AR}^\rho )} \right) \end{aligned}$$

is lower-semicontinuous and convex in $\rho _A \in {\mathcal {S}}({\textsf{A}})$ for each $\alpha \ge 1$ (where $\psi _{AR}^\rho$ is a purification of $\rho _A$), and upper-semicontinuous and concave in $\alpha \in [1,2]$ for each $\rho _A \in {\mathcal {S}}({\textsf{A}})$ Moreover, under the finite-dimension assumption, the sets ${\mathcal {S}}({\textsf{A}})$ and [1, 2] are both convex and compact, and the convex-concave objective function is always finite and bounded. Hence, the assertion of the saddle-point with its saddle-value being the error-exponent function of channel ${\mathscr {N}}_{A\rightarrow B_1 B_2\ldots B_L}$ follows from [114, Theorem 36.3].

Next, we prove Item (iv). Note that the error-exponent function is given by the sandwiched Rényi information. We will slightly weaken it via the Petz’s Rényi information [110, 112, 130]. It will still lead to our goal since both the two Rényi information quantities have the same limiting behavior as $\alpha \rightarrow 1$. Such a relaxation will simplify the analysis due to the closed-form expression of Petz’s Rényi information [104, 110, 131]. Moreover, we prove the unipartite case (i.e. $L=1$) as follows; the multipartite case for general $L\in \mathbb {N}$ follows straightforwardly.

For each $n\in \mathbb {N}$, by Proposition 2-(iii), we let $(\rho _n,\alpha _n) \in {\mathcal {S}}({\textsf{A}})\times [1,2]$ be a saddle-point of the function

A.26 $$\begin{aligned} (\rho ,\alpha )\mapsto \frac{\alpha -1}{\alpha } \left( r_n - I_{\alpha _n}(B:R)_{{\mathscr {N}}(\psi _{AR}^\rho )} \right) , \end{aligned}$$

such that $\psi _n = \psi _{AR}^\rho$ is a purification of $\rho _{A}$, and

A.27 $$\begin{aligned} E_{r_n}({\mathscr {N}})&= \frac{\alpha _n-1}{\alpha _n} \left( r_n - I_{\alpha _n} (B:R)_{{\mathscr {N}}(\psi _n)} \right) \end{aligned}$$

A.28 $$\begin{aligned}&\ge \frac{\alpha -1}{\alpha } \left( r_n - I_{\alpha }(B:R)_{{\mathscr {N}}(\psi _n)} \right) , \forall \, \alpha \in [1,2]. \end{aligned}$$

To ease the burden of notation, let us denote

A.29 $$\begin{aligned} I&\,{:=}\, I({\mathscr {N}}), \end{aligned}$$

A.30 $$\begin{aligned} I_n&\,{:=}\, I(B:R)_{{\mathscr {N}}(\psi _n)}, \end{aligned}$$

A.31 $$\begin{aligned} V_n&\,{:=}\, V(B:R)_{{\mathscr {N}}(\psi _n)}. \end{aligned}$$

Note that since $r_n \rightarrow I$, by Proposition 2-(i), we must have

A.32 $$\begin{aligned} \lim _{n\rightarrow \infty } \alpha _n = 1. \end{aligned}$$

On the other hand,

A.33 $$\begin{aligned} I_{\alpha _n}(B:R)_{{\mathscr {N}}(\psi _n)} \ge I_{\alpha _n}(B:R)_{{\mathscr {N}}(\psi )}, \quad \forall \, \psi _{AR}. \end{aligned}$$

This guarantees that $\psi _n$ converges to some (channel-capacity achieving) pure state, say $\psi _\infty$, that satisfies

A.34 $$\begin{aligned} \lim _{n\rightarrow \infty } I_{\alpha _n}(B:R)_{{\mathscr {N}}(\psi _n)} = I(B:R)_{{\mathscr {N}}(\psi _\infty )} = I. \end{aligned}$$

We introduce the Petz-type quantities [130]:

A.35 $$\begin{aligned} \bar{D}_\alpha (\rho \,\Vert \, \sigma )&\,{:=}\, \frac{1}{\alpha -1}\log {{\,\textrm{Tr}\,}}\left[ \rho ^\alpha \sigma ^{1-\alpha }\right] , \end{aligned}$$

A.36 $$\begin{aligned} \bar{I}_\alpha (B:R)_{{\mathscr {N}}(\psi )}&\,{:=}\, \inf _{\sigma _{R}\in {\mathcal {S}}({\textsf{R}})} \bar{D}_\alpha \left( {\mathscr {N}}_{A\rightarrow B} (\psi _{AR}) \,\Vert \, {\mathscr {N}}_{A\rightarrow B} (\psi _{A}) \otimes \sigma _R \right) . \end{aligned}$$

(One can also work with a Rényi information without minimizing $\sigma _{R}$ [85, 104] since they all have the same Taylor’s series expansion aroudn $\alpha = 1$.)

Now, we are all set for the proof. From Eq. (A.28), we choose a specific

A.37 $$\begin{aligned} \bar{\alpha }_n \,{:=}\, 1 + \frac{I-I_n+a_n}{V_n}, \end{aligned}$$

to replace $\alpha _n$, which yields a lower bound to the error-exponent function of channel. We calculate

A.38 $$\begin{aligned} E_{r_n}({\mathscr {N}})&= \frac{\alpha _n-1}{\alpha _n} \left( r_n - I_{\alpha _n}(B:R)_{{\mathscr {N}}(\psi _n)} \right) \end{aligned}$$

A.39 $$\begin{aligned}&\ge \frac{I-I_n+a_n}{V_n\left( 1 + \frac{I-I_n+a_n}{V_n} \right) } \left( I + a_n - I_{\bar{\alpha }_n}(B:R)_{{\mathscr {N}}(\psi _n)} \right) \end{aligned}$$

A.40 $$\begin{aligned}&\overset{\text {(a)}}{\ge } \frac{I-I_n+a_n}{V_n\left( 1 + \frac{I-I_n+a_n}{V_n} \right) } \left( I + a_n - \bar{I}_{\bar{\alpha }_n}(B:R)_{{\mathscr {N}}(\psi _n)} \right) \end{aligned}$$

A.41 $$\begin{aligned}&\overset{\text {(b)}}{=} \frac{I-I_n+a_n}{V_n\left( 1 + \frac{I-I_n+a_n}{V_n} \right) } \left( I + a_n - I_n - \frac{I - I_n + a_n}{2} - \frac{(\bar{\alpha }_n-1)^2}{2}{\mathfrak {R}}_n \right) , \end{aligned}$$

A.42 $$\begin{aligned}&= \frac{(I-I_n+a_n)^2}{2V_n} \frac{1}{1+\frac{I-I_n+a_n}{V_n}} \left( 1 - \frac{\bar{\alpha }_n-1}{V_n} {\mathfrak {R}}_n \right) \end{aligned}$$

A.43 $$\begin{aligned}&\overset{\text {(c)}}{\ge } \frac{a_n^2}{2V_n} \frac{1}{1+\frac{I-I_n+a_n}{V_n}} \left( 1 - \frac{\bar{\alpha }_n-1}{V_n} \Upsilon \right) , \end{aligned}$$

where (a) is because $D_\alpha \le \bar{D}_\alpha$ for all $\alpha \ge 1$ [132, 129, Proposition 3.20]; and in (b) we invoke Taylor’s series expansion of the map $\alpha \mapsto \bar{I}_{\alpha }(B:R)_{{\mathscr {N}}(\psi )}$ at $\alpha =1$ [78, 85, 133]:

A.44 $$\begin{aligned} \bar{I}_{\bar{\alpha }_n}(B:R)_{{\mathscr {N}}(\psi _n)}&= I_n + \frac{\bar{\alpha }_n-1}{2} V_n + \frac{(\bar{\alpha }_n-1)^2}{2} {\mathfrak {R}}_n, \end{aligned}$$

A.45 $$\begin{aligned} {\mathfrak {R}}_n&\,{:=}\, \left. \frac{\partial ^2}{ \partial \alpha ^2 } \bar{I}_{{\alpha }}(B:R)_{{\mathscr {N}}(\psi _n)}\right| _{\alpha = {\tilde{\alpha }}_n \in [1,\bar{\alpha }_n] }. \end{aligned}$$

The last line (c) is due to the fact that $I - I_n\ge 0$ for all $n\in \mathbb {N}$, and

A.46 $$\begin{aligned} \Upsilon \,{:=}\, \max _{ (\psi ,\alpha ) \in {\mathcal {S}}({\textsf{A}}\otimes {\textsf{R}}) \times [1,2] } \left| \frac{\partial ^2}{ \partial \alpha ^2 } I_{{\alpha }}(B:R)_{{\mathscr {N}}(\psi )}\right| . \end{aligned}$$

As already pointed out in [85], such a factor is finite by the finite-dimensional assumption of the underlying Hilbert space ${\textsf{A}}$, ${\textsf{B}}$, and ${\textsf{R}}$, the uniform continuity of the quantity in (A.46), which holds because of the closed-form expression of the Petz’s Rényi information [104, 110, 131].

Note that $\bar{\alpha }_n \rightarrow 1$ by Eq. (A.34). We have

A.47 $$\begin{aligned} \liminf _{n\rightarrow \infty }\frac{E_{r_n}({\mathscr {N}})}{a_n^2}&\ge \liminf _{n\rightarrow \infty } \frac{1}{2V(B:R)_{{\mathscr {N}}(\psi _n)}} \end{aligned}$$

A.48 $$\begin{aligned}&\ge \frac{1}{2V({\mathscr {N}})}, \end{aligned}$$

where the last line follows from the continuity of $\psi \mapsto V(B:R)_{{\mathscr {N}}(\psi )}$ [85, 134], the fact that $\psi _n \rightarrow \psi _\infty$ and Eq. (A.34), and the definition of $V({\mathscr {N}})$ given in Eq. (2.16). We conclude the proof $\square$

## Appendix B. The Post-Selection Technique

The following facts are the well-known *Post-Selection Technique* [69] also known as *de Finetti reductions* or *universal state* [135], and the formulation below is taken from [3, Appendix D].

### Fact 2

([69]) Let $\varepsilon >0$ and ${\mathcal {E}}^{n}_{A}$ and ${\mathcal {F}}^{n}_{A}$ be completely positive and trace-preserving maps from ${\mathcal {B}}({\textsf{A}}^{\otimes n})$ to ${\mathcal {B}}({\textsf{B}})$. If there exists a CPTP map $K_{\pi }$ for any permutation $\pi$ such that $({\mathcal {E}}^{n}_{A}-{\mathcal {F}}^{n}_{A})\circ \pi =K_{\pi }\circ ({\mathcal {E}}^{n}_{A}-{\mathcal {F}}^{n}_{A})$, then ${\mathcal {E}}^{n}_{A}$ and ${\mathcal {F}}^{n}_{A}$ are $\varepsilon$-close in diamond norm whenever

B.1 $$\begin{aligned} \left\| (({\mathcal {E}}^{n}_{A}-{\mathcal {F}}^{n}_{A})\otimes {\mathcal {I}}_{RR'})(\zeta ^{n}_{ARR'})\right\| _{1}\le \varepsilon (n+1)^{-(|{\textsf{A}}|^{2}-1)} , \end{aligned}$$

where $\zeta ^{n}_{ARR'}$ is a purification of the de Finetti state $\zeta _{AR}^{n}=\int \psi _{AR}^{\otimes n}\, \textrm{d}(\psi _{AR})$ with $\psi _{AR}$ is a pure state on ${\textsf{A}}\otimes {\textsf{R}}$ with ${\textsf{A}}\cong {\textsf{R}}$, and $\textrm{d}(\cdot )$ is the measure on the normalized pure states on ${\textsf{A}}\otimes {\textsf{R}}$ induced by the Haar measure on the unitary group acting on ${\textsf{A}}\otimes {\textsf{R}}$, normalized to $\int \textrm{d}(\cdot )=1$. Furthermore, we can assume without loss of generality that $|{\textsf{R}}'|\le (n+1)^{|{\textsf{A}}|^{2}-1}$.

By Carathéodory’s theorem for convex hulls, one also has

### Fact 3

([136]) Let $\zeta _{AR}^{n}=\int \psi _{AR}^{\otimes n}\, \textrm{d}(\psi _{AR})$ as in Fact 2. Then, we can write $\zeta _{AR}^{n}=\sum _{i}p_{i}\left( \psi ^{i}_{AR}\right) ^{\otimes n}$ with $\omega ^{i}_{AR}$ being a pure state on ${\textsf{A}}\otimes {\textsf{R}}$, $i\in \{1,2,\ldots ,(n+1)^{2|{\textsf{A}}||{\textsf{R}}|-2}\}$, and $\{p_{i}\}_i$ a probability distribution.

## Footnotes

1 Of course the base station can send information to each user equipment individually. This amounts to a point-to-point communication with the *Time-Division Multiple Access* scheme, which is a special case of the *Time Sharing* [47, Proposition 4.1]. However, when there are enormous users equipments involved in the network setting, such a method is considerably time inefficient. Hence, as referred to a network setting in this paper, we demand the sender and receivers to adopt simultaneous encoding and decoding strategies.

2 Even though the term “single-letter characterization” for a networking information-theoretic task is debatable [52, 53], whether Marton’s inner bound is optimal is—to our best knowledge—still open [47, 54–60].

3 Our approach actually holds for a more stronger notion of *coherent feedback simulation*; see Remark 8.

4 A specialized version of the bipartite convex splitting with quantum relative entropy as an error criterion was introduced in [66, Lemma 2]. We refer the readers to Sect. 1.2 for discussions and comparisons.

5 Technically speaking, we do not employ the *smooth entropy framework*; instead, we use the interpolation technique as in the unipartite setting [72, 79]. Hence, what we avoid should be termed as *simultaneous interpolation*.

6 We remark that the time-sharing technique requires *synchronization* between the sender and receivers [47, Remark 4.3]. Such a requirement can be practically demanding when the there is a large number of users in the network.

7 The non-coherent Quantum State Merging without time sharing was investigated in [90, §V]. However, we are not able to confirm the correctness for the case of more than 2 senders.

8 The authors termed [66, Lemma 2] as the “tripartite” convex-split lemma. However, we intend to call it a “bipartite” convex splitting for the following reason. The unipartite convex splitting [70, 72] aims to decouple systems *A* and *E*, while the bipartite convex splitting aims to decouple systems *A* into two parts, i.e. systems *A* and *B* in Eq. (1.4). Our terminology is also consistent with the classical convex-split lemma used in [71, Fact 7].

9 Note that our definition of channel sandwiched Rényi information given in Eq. (2.5) is different from the one given in [103, §7] wherein the authors considered $I_\alpha ({\mathscr {N}}_{A\rightarrow B}) \,{:=}\, \sup _{\psi _{AR}} I_\alpha (R:B)_{({\mathscr {N}}_{A\rightarrow B}\otimes \textrm{id}_R)(\psi )}$, with *B* and *R* swapped comparing to (2.5). The two definitions coincide as $\alpha = 1$.

10 We note that sometimes the multipartite quantum mutual information is defined in an alternative way, e.g. $I(A:B:C)_\rho \,{:=}\, I(A: B)_\rho + I(A: C)_\rho - I(A: BC)_\rho$. However, we will adopt the definition in Eq. (2.12) in this paper.

11 We note that the concavity established in [110] is for a special case that system *A* is classical and $\tau _{A} = \rho _{A}$. However, the same reasoning applies here as well.

12 If *Y* is not invertible, we then take the Moore-Penrose pseudo-inverse of *Y* instead.
